# Developing a Toolbox of Antibodies Validated for Array Tomography-Based Imaging of Brain Synapses

**DOI:** 10.1523/ENEURO.0290-23.2023

**Published:** 2023-12-08

**Authors:** Kristina D. Micheva, Belvin Gong, Forrest Collman, Richard J. Weinberg, Stephen J. Smith, James S. Trimmer, Karl D. Murray

**Affiliations:** 1 Department of Cellular and Molecular Physiology, Stanford School of Medicine, Stanford, 94305, CA; 2 Department of Physiology and Membrane Biology, University of California Davis School of Medicine, Davis, 95618, CA; 3 Allen Institute for Brain Science, Seattle, 98109, WA; 4 Department of Cell Biology and Physiology, University of North Carolina, Chapel Hill, 27514, NC; 5 Department of Psychiatry and Behavioral Sciences, University of California, Davis, Davis, 95618, CA

**Keywords:** connectivity, electron microscopy, nanoscale, proteome, synaptome, ultrastructure

## Abstract

Antibody (Ab)-based imaging techniques rely on reagents whose performance may be application specific. Because commercial antibodies are validated for only a few purposes, users interested in other applications may have to perform extensive in-house antibody testing. Here, we present a novel application-specific proxy screening step to efficiently identify candidate antibodies for array tomography (AT), a serial section volume microscopy technique for high-dimensional quantitative analysis of the cellular proteome. To identify antibodies suitable for AT-based analysis of synapses in mammalian brain, we introduce a heterologous cell-based assay that simulates characteristic features of AT, such as chemical fixation and resin embedding that are likely to influence antibody binding. The assay was included into an initial screening strategy to generate monoclonal antibodies that can be used for AT. This approach simplifies the screening of candidate antibodies and has high predictive value for identifying antibodies suitable for AT analyses. In addition, we have created a comprehensive database of AT-validated antibodies with a neuroscience focus and show that these antibodies have a high likelihood of success for postembedding applications in general, including immunogold electron microscopy. The generation of a large and growing toolbox of AT-compatible antibodies will further enhance the value of this imaging technique.

## Significance Statement

Array tomography (AT) is a powerful volume microscopy technique for high-dimensional analysis of complex protein populations in cells and organelles, including synapses. AT involves the use of ultrathin serial sections embedded in resin and subjected to multiple rounds of immunofluorescence antibody (Ab) labeling and imaging. AT relies on antibody-based detection of proteins but because commercial antibodies are typically validated for other applications they often fail for AT. To identify antibodies with high probability of success in AT we developed a novel screening strategy and used this to create a comprehensive database of AT-validated antibodies for neuroscience.

## Introduction

Array tomography (AT) is a powerful technique for the analysis of large populations of synapses with deep proteomic dimensionality. AT involves preparing ultrathin serial sections from brain tissue that has been embedded in acrylic resin, and subjecting this array of sections to multiplex immunofluorescence antibody (Ab) labeling and imaging, followed by multiple rounds of iterative Ab removal, reprobing, and imaging (Micheva and Smith, 2007). After many rounds of imaging, sections can be exposed to heavy metal stains, and further imaged with scanning electron microscopy. Ultimately, images are reconstructed into three-dimensional volumes of brain ultrastructure with fluorescent labeling overlays (Collman et al., 2015). This technique can simultaneously interrogate the proteomic composition of thousands of synapses with deep dimensionality (Micheva et al., 2010a; O’Rourke et al., 2012; Holderith et al., 2020). Unfortunately, many commercial Abs do not exhibit efficacy and/or specificity when applied to brain samples prepared for AT (Micheva and Smith, 2007; Micheva et al., 2010a), hindering efforts to broadly implement this powerful imaging technique.

While further refinement of tissue preparation for AT could potentially lead to improved labeling with existing antibodies, such efforts are severely limited by two considerations. First, because multiplexing is a major advantage of the method, one needs to find conditions that will be beneficial for all antibodies. Often, changing one parameter (e.g., less fixation) may improve the performance of an antibody, while decreasing the performance of other antibodies, or resulting in loss of smaller cytosolic antigens and thus hindering their detection. Second, the ability to preserve ultrastructure and use both immunofluorescence and electron microscopy is a key feature of AT. Antigenicity can be improved by resin removal (Holderith et al., 2020), but this damages the ultrastructure, making it difficult to examine the tissue under the electron microscope (Brorson and Reinholt, 2008). Therefore, we focused our efforts on generating and validating a set of Abs with high efficacy and specificity for brain tissue prepared using current AT protocols.

We had previously developed a reliable pipeline for generating, screening and validating monoclonal antibodies (mAbs) for neuroscience research, initially focusing on voltage-gated potassium channels (Bekele-Arcuri et al., 1996). This approach comprised analyses of numerous candidate mAbs in immunoblot and immunohistochemistry assays against mammalian brain samples (Bekele-Arcuri et al., 1996). This system reliably yielded mAbs against other ion channels (Boiko et al., 2001), synaptic scaffolds (Tiffany et al., 2000; Rasband et al., 2002), adhesion molecules (Rasband et al., 2001; Rasband and Trimmer, 2001) neurotransmitter receptors (Perez-Otano et al., 2001), and a variety of other targets. This approach was used to provide highly validated mAbs to the research community in an National Institutes of Health (NIH)-funded effort at the University of California Davis/NIH NeuroMab Facility (Rhodes and Trimmer, 2006; Gong et al., 2016). A key aspect of Ab validation is to test them for efficacy and specificity directly in the particular application, sample type and under the exact sample preparation and labeling conditions in which they will be subsequently used (Rhodes and Trimmer, 2006; Lorincz and Nusser, 2008; Bordeaux et al., 2010; Gong et al., 2016). When a new immunolabeling technique like AT is introduced to the scientific community, it remains uncertain whether existing Ab collections will be effective and specific in the new application. Initial tests on a set of commercial Abs suggested that only a restricted subset of Abs screened on conventional assays would show efficacy and specificity for ultrathin sections embedded in plastic. Accordingly, identifying which Abs can be used on AT samples for systematic evaluation of brain synapses remains a requirement for broad and effective use of this powerful technique.

Here, we describe efforts aimed at developing a reliable platform for validating Abs for AT. We present the results of using this platform in analyses of existing mAbs developed and/or validated for other purposes, and in new projects specifically aimed at developing novel mAbs for use in AT.

## Materials and Methods

### Hybridoma generation and conventional mAb screen

Mouse immunizations, splenocyte isolation, hybridoma generation and conventional screening were performed following the protocols in Bekele-Arcuri et al., 1996; and Gong et al., 2016; except that electrofusion was used to generate hybridomas. Two ELISA assays, one against purified protein immunogen, and one against transfected heterologous cells overexpressing the full-length target protein, were used in parallel as the primary screen (Gong et al., 2016). A selected set of ELISA-positive candidates were taken through subsequent screens, including immunocytochemistry on transfected cells, immunoblots on brain subcellular fractions, and immunohistochemistry on conventionally prepared brain sections (Bekele-Arcuri et al., 1996; Rhodes and Trimmer, 2008; Gong et al., 2016).

### Preparation of cell pellet arrays for AT cell-based proxy screen

The cell-based proxy screen (CBS) was developed from a previously reported protocol for preparing cultured cells for transmission electron microscopy (Schrand et al., 2010). Briefly, COS-1 cells were cultured overnight in 10-cm tissue culture plates until a confluency of ∼70% and then transfected with mammalian expression plasmids using Lipofectamine 2000 (ThermoFisher, catalog #11668030) per manufacturer’s instructions. Cells were either co-transfected with plasmids encoding enhanced green fluorescent protein (EGFP) and the target protein of interest, or with plasmid encoding the target protein fused to a reporter tag (EGFP, FLAG). Transfected cells were incubated at 37°C/5% CO_2_ for 72 h, then harvested in Versene with manual pipetting to release adherent cells. Cells from multiple culture plates were pooled into a single 15-ml tube and centrifuged at 1000 × *g* for 5 min at room temperature (RT). The subsequent pellet was transferred to a glass vial and fixed for 2 h at RT in AT fixative [4% formaldehyde (FA) in 10 mm PBS (138 mm NaCl, 2.7 mm KCl) with 2.5% sucrose, made fresh from 8% aqueous FA; Electron Microscopy Sciences (EMS), catalog #157-8)]. The pellet was rinsed three times for 10 min each in PBS containing 50 mm glycine, followed by dehydration using 5-min incubations in solutions of 50% ethanol (1×) and 70% ethanol (3×). The pellet was then washed twice for 5 min each in a solution of 3 parts LR White acrylic resin (hard grade, SPI supplies catalog # 2645) and 1 part 70% ethanol, and then four times for 5 min each in 100% LR White at 4°C. The pellet was left in LR White overnight at 4°C, then transferred to a gelatin capsule filled with LR White resin, capped, and incubated for 24 h at 55°C. To generate semi-thin (400 nm) sections, the plastic “bullet” containing embedded cells was manually trimmed and then sectioned on an ultramicrotome (Leica, Ultracut UCT). Sections were collected using a thin metal loop, placed in single wells of a collagen-coated glass bottom 96-well plate (Corning 4582), air dried and stored in the dark at RT until screening.

### Immunolabeling and analyses of CBS proxy assay

Semi-thin (400 nm) sections in 96-well plates were rinsed in 50 mm glycine in Tris-buffered saline (TBS; 50 mm Tris, 150 mm NaCl, pH 7.6) for 5 min at RT. Glycine was removed and sections were incubated in blocking buffer [0.05% Tween 20 (EMS, catalog #25564) and 0.1% BSA (EMS, catalog # 25 557) in TBS] for 5 min at RT and then incubated in primary Ab in blocking buffer for 2 h at RT. Following three washes in TBS for 5 min each, sections were incubated in goat anti-mouse IgG secondary Ab conjugated to Alexa Fluor-594 for 30–45 min at RT. Following secondary labeling, sections were washed in TBS for 5 min each in RT. Sections were imaged using a 40×/1.2 NA objective on a Zeiss AxioObserver Z1 microscope with an AxioCam HRm digital camera controlled with Axiovision software (Zeiss). Target labeling of Ab was assessed by comparing fluorescent signal in red (Alexa Fluor-594) and green (EGFP) channels for degree of colocalization (specificity of target label) and for labeling intensity. Labeling was rated on a scale of 0 (no label) to 4 (intense and complete colocalization).

### Preparation of arrays for AT from mouse neocortex

Arrays were prepared following the protocol described previously (Micheva et al., 2010b). All animal procedures were performed in accordance with the Administrative Panel on Laboratory Animal Care at Stanford University. Briefly, pentobarbital anesthetized mice were subjected to intracardial perfusion with 4% FA in PB (0.1 m phosphate buffer, pH 7.4) made fresh from powdered paraformaldehyde. Following removal of the perfusion-fixed brain, small (1 mm^3^) blocks of cerebral cortex were dissected and immediately transferred to AT fixative for 1 h at RT followed by overnight incubation at 4°C. Tissue blocks were then washed, dehydrated, and embedded in LR White resin according to steps described above for CBS pellets. After embedding, blocks were manually trimmed, and serial sections (70 nm) were cut with an ultramicrotome (Leica, Ultracut UCT) and collected onto gelatin-coated glass coverslips. Sections were air dried and stored in the dark at RT until ready to be labeled.

### Human neocortical tissue preparation for AT

Fresh human tissue and autopsy tissue prepared for AT from previous studies was used (Micheva et al., 2018). Briefly, human cortical samples were rinsed in saline and placed in RT fixative (4% FA in PB) for 1 h. The tissue was further fixed for 23 h at 4°C, for a total time of 24 h in fixative. The tissue was then transferred to PBS with 0.01% sodium azide and stored at 4°C before further processing. The tissue was dehydrated and embedded following the same protocol as for the CBS pellet, except that incubation times in the different ethanol solutions and in resin were increased to 10 min.

### Lowicryl HM20 embedding for conjugate immunofluorescence-SEM AT applications

All animal procedures were performed in accordance with the University of North Carolina animal care committee’s regulations. After deep anesthesia with pentobarbital, adult mice (three to four months old) were perfusion-fixed with a mixture of 2% glutaraldehyde/2% FA, dissolved in 0.1 m phosphate buffer (pH 6.8). Brains were removed and postfixed overnight at 4°C in the same fixative. Following extensive washes in buffer, 200-μm-thick Vibratome sections were collected, incubated on ice on a shaker with 0.1% CaCl_2_ in 0.1 m sodium acetate for 1 h, then cryoprotected through 10% and 20% glycerol, and overnight in 30% glycerol in sodium acetate solution. The next day, small tissue chunks from neocortex were dissected out and quick-frozen in a dry ice/ethanol bath. Freeze-substitution was performed using a Leica AFS instrument with several rinses in cold methanol followed by substitution in a 2–4% solution of uranyl acetate in methanol, all at −90°C. After 30-h incubation, the solution was slowly warmed to −45°C and infiltrated with Lowicryl HM20 over 2 d. Capsules containing tissue chunks were then exposed to UV and gradually warmed to 0°C. Polymerized capsules were removed from the AFS apparatus and further exposed to UV at RT for an additional day, to complete curing of the plastic.

### Immunofluorescent labeling and analysis of brain AT sections

Ultrathin (70 nm) sections on coverslips were incubated in 50 mm glycine in TBS for 5 min at RT, followed by blocking solution (0.05% Tween 20 and 0.1% BSA in TBS) for 5 min at RT, and then incubated in primary Abs diluted in blocking solution overnight at 4°C. The reference antibodies are listed in Table 1. Following three washes in TBS for 5 min each, sections were incubated in cross-adsorbed Alexa Fluor dye-conjugated goat secondary Abs (ThermoFisher Scientific), diluted 1:150 in blocking solution for 30 min at RT. The mAbs were detected using Alexa Fluor-594-conjugated goat anti-mouse IgG (H+L; Invitrogen catalog #A-11032) and the reference Ab with an Alexa Fluor-488-conjugated goat Ab against the appropriate host (Invitrogen, catalog #A-11034 anti-rabbit, catalog #A-11073 anti-guinea pig or catalog #A-11039 anti-chicken). Subsequently, labeled sections were washed three times in TBS for 5 min each, followed by three rinses in water for 30 s each. Coverslips with sections were mounted onto glass slides using SlowFade Gold Antifade mountant with DAPI (Invitrogen #S36964) and imaged the same day using a 63×/1.4 Plan-Apochromat 1.4 NA oil objective on a Zeiss AxioImager Z1 microscope with an AxioCam HR digital camera controlled with Axiovision software (Zeiss). Image ZVI files were converted to TIFF and uploaded into Fiji Imaging software for analysis. Images of multiplex labeling from at least three serial sections were aligned using the DAPI signal with the MultiStackReg plugin in FIJI (Thévenaz et al., 1998; Schindelin et al., 2012) and immunolabeling was assessed for proper localization against a reference marker. Quality of labeling was assessed by experienced observers and rated on a scale of 0 (no label or off target only) to 4 (target only label).

**Table 1:** List of reference antibodies used for antibody screening

Antigen	Host	Antibody source	Dilution	RRID
Synaptic				
Synapsin-1	Rabbit	Cell Signaling 5297	1:500	AB_2616578
Excitatory synapses				
VGluT1	Guinea pig	Millipore AB5905	1:5000	AB_2301751
PSD95	Rabbit	Cell Signaling 3450	1:200	AB_2292883
Inhibitory synapses				
GABA^*^	Guinea pig	Millipore AB175	1:5000	AB_91011
GAD2	Rabbit	Cell Signaling 5843	1:200	AB_10835855
Gephyrin	Mouse	NeuroMab 75-465, clone L106/4	1:100	AB_2716264

* Requires glutaraldehyde in the fixative. RRID, Research Resource Identifier.

### Synaptic antibody characterization tool (SACT) analysis

To identify top Ab candidates for synaptic target localization we used the SACT program (Simhal et al., 2018), which applies an unsupervised probabilistic detection algorithm (Simhal et al., 2017) to identify fluorescent puncta and determine whether they are located at synapses. For each candidate Ab, the size, volume, and density of immunolabeled puncta was measured and compared with similar measures made using an AT synaptic marker reference Ab in the same sections (Synapsin-1 or PSD-95). To evaluate the sensitivity and specificity of a candidate Ab we plotted the target synaptic density of each candidate (defined as the number of synapses detected with the candidate Ab per unit volume) and the target specificity ratio (TSR), defined as the number of synapses detected by the candidate Ab relative to the total number of Ab puncta (Simhal et al., 2018).

### Immunogold labeling of osmium-treated samples

For immunogold EM of osmium-treated tissue embedded in LR White, the samples were prepared similarly to Immunofluorescence AT, except that the fixative contained 0.1% glutaraldehyde in addition to the 4% FA, and a postfixation step was added with osmium tetroxide (0.1%) and potassium ferricyanide (1.5%) with rapid microwave irradiation (PELCO 3451 laboratory microwave system with ColdSpot; Ted Pella), three cycles of 1 min on–1 min off–1 min on at 100 W, followed by 30 min at RT. The immunolabeling protocol was similar to the immunofluorescence labeling, with two additional steps in the beginning: treatment for 1 min with saturated sodium metaperiodate solution in dH_2_O (to remove osmium) and 5 min with 1% sodium borohydride in Tris buffer to reduce free aldehydes resulting from the presence of glutaraldehyde in the fixative. A 10-nm gold-labeled goat anti-mouse IgG secondary Ab (SPI Supplies) was used at 1:25 for 1 h. After washing off the secondary Ab, the sections were treated with 1% glutaraldehyde for 1 min to fix the Abs in place. The sections were poststained with 5% uranyl acetate for 30 min and lead citrate for 1 min.

### Immunogold labeling of Lowicryl HM20-embedded tissue

Thin sections (∼80 nm) of adult mouse cortex embedded in Lowicryl HM20 were cut and collected on nickel mesh grids. Grids were blocked in 1% bovine serum albumin in Tris-buffered saline pH 7.6 with 0.005% Tergitol NP-10, and incubated overnight at 21–24°C with the primary Ab. Grids were then rinsed, blocked in 1% normal goat serum in Tris-buffered saline pH 8.2, and incubated in goat anti-mouse secondary Abs conjugated to 10- or 20-nm-diameter gold particles (Ted Pella). Grids were counterstained with 1% uranyl acetate, followed by Sato’s lead, and examined in a Philips Tecnai transmission electron microscope at 80 KV, and images collected with a 1024 × 1024 cooled CCD (Gatan).

## Results

### Efficacy and specificity of commercial Abs against synaptic proteins for array tomography

**Figure 1.**
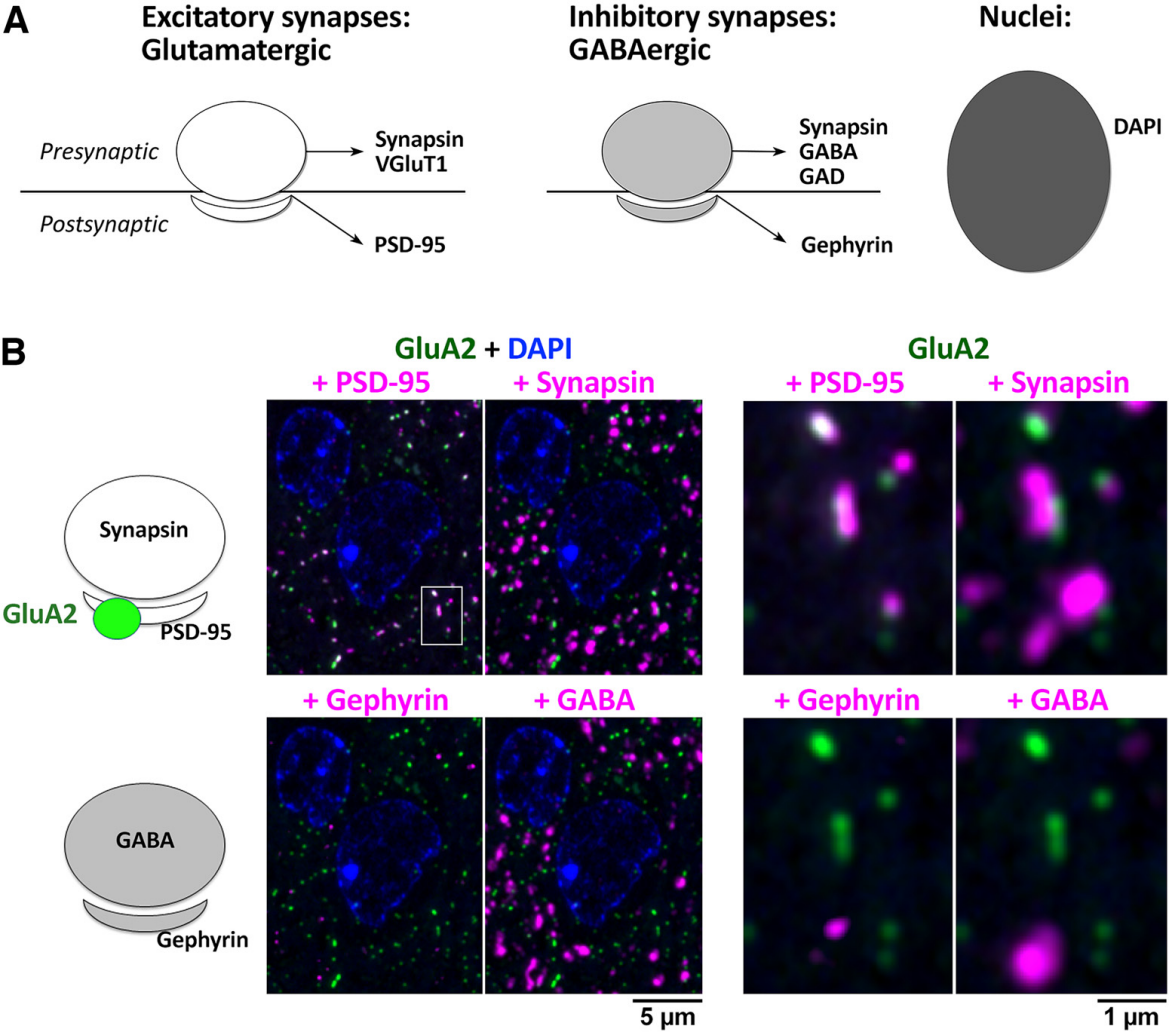
Initial AT evaluation strategy for identifying synaptic Abs validated in other applications. ***A***, Common reference markers for presynaptic and postsynaptic locations and nuclei. ***B***, Example evaluation of an Ab against GluA2 (Abcam ab206293), a glutamatergic receptor with a known postsynaptic localization at excitatory synapses. A single 70-nm section from adult mouse cortex, labeled with the GluA2 Ab (green) and synaptic markers PSD-95 (Cell Signaling 3450), synapsin (Synaptic Systems 106006), gephyrin (NeuroMab L106/93), GABA (Millipore AB175), and the nuclear label DAPI. The panel to the right is an enlarged view of the boxed area in the left panel. The GluA2 Ab was scored as excellent, based on its colocalization with PSD-95, adjacency to synapsin and minimal background label. See Extended Data Figures 1-1 and 1-2 for more details.

Initial efforts to identify AT-appropriate Abs that successfully labeled target proteins in sections from FA-fixed and LR White embedded mouse neocortex relied on ad hoc sampling of the vast array of preexisting Abs from commercial sources. Over 300 commercial Abs, selected based on available literature and personal communication, were evaluated for efficacy and specificity for AT (Extended Data Fig. 1-1). Criteria for success included immunolabeling that matched known cellular expression and subcellular localization of the target protein. When synaptic proteins were targeted, the subcellular distribution of the immunofluorescence of the tested Ab was evaluated by assessing colocalization with well-known reference synaptic Abs (Fig. 1; Table 1) and other AT validated antibodies (Extended Data Fig. 1-2). All of the reference antibodies were characterized extensively and used in several previously-published studies (Micheva et al., 2010a; Collman et al., 2015; Simhal et al., 2018). Potential background or nonspecific labeling was evaluated using “exclusion” markers defined for each target protein; for example, inhibitory synapse markers when testing Abs against proteins thought to be restricted to excitatory synapses. The labeling pattern of each Ab was compared with that for the nuclear marker DAPI to control for background nuclear immunolabeling (Fig. 1). Scoring was performed using visual inspection of images by a trained observer. We found that even with widely-used commercial antibodies generally considered to yield optimal results, up to 50% fail completely (139/306 tested; Extended Data Fig. 1-1). Even more alarming was the observation that for 32% of the targets (63 out of 196) we failed to identify an AT-suitable commercial antibody. Therefore, we set out to design a more focused and application-specific screening process.

10.1523/ENEURO.0290-23.2023.f1-1 Extended Data Figure 1-1 Table of all antibodies tested for AT. RRID, Research Resource Identifier. “Tested In” column shows the species where the antibody was tested. M, mouse; H, human; R, rat; Z, zebrafish. The results from the antibody testing indicated in the next column, “Target Label,” apply to all species where the antibody was tested, unless explicitly stated. Testing was performed on formaldehyde fixed tissue embedded in LR White, except for the antibodies indicated with *, which require glutaraldehyde in the fixative. The last column, “Performance on Lowicryl,” indicates results of the testing on tissue fixed with a combination of formaldehyde and glutaraldehyde and embedded in Lowicryl HM20. Download Figure 1-1, XLS file.

**Figure 2.**
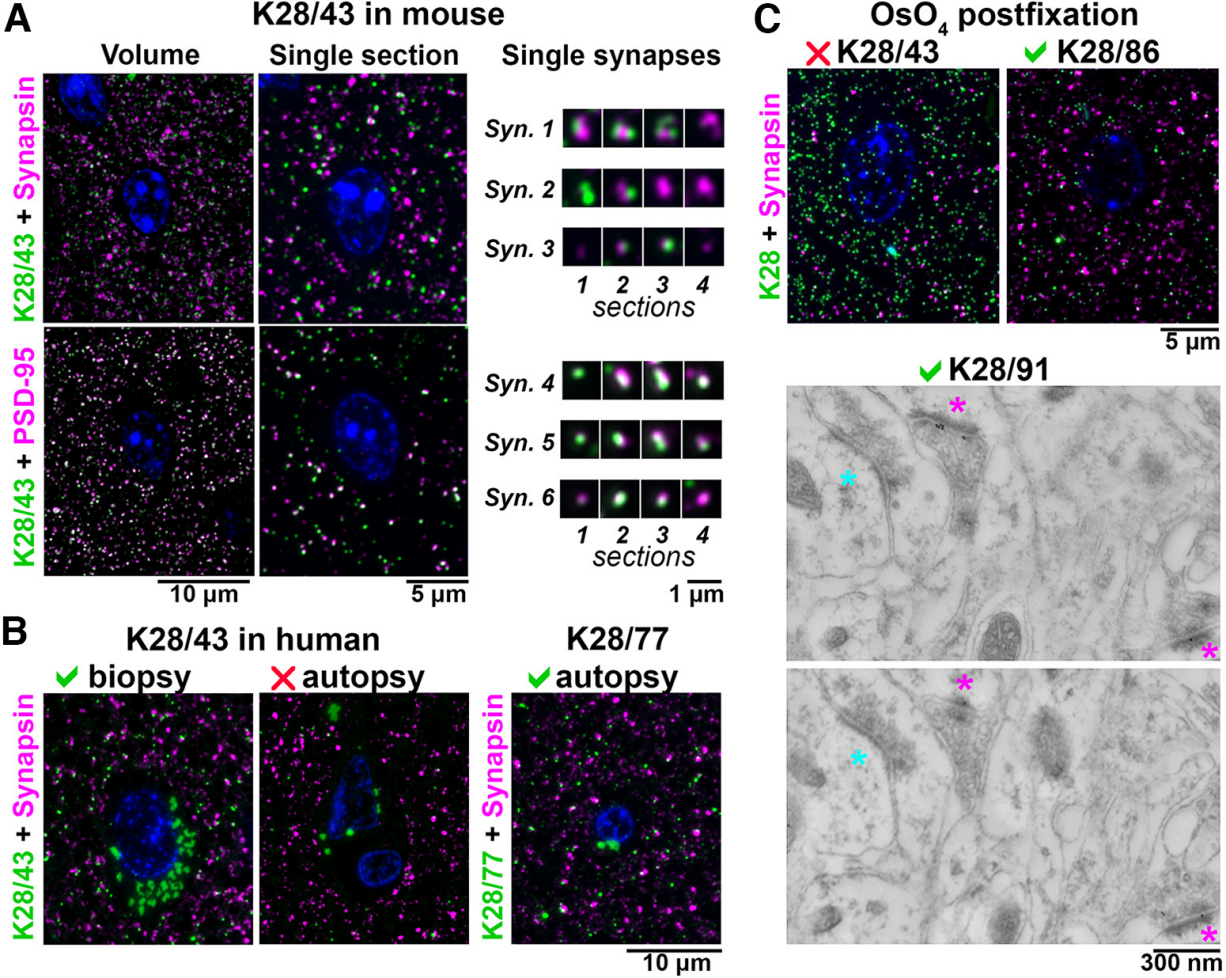
Application-specific performance of mAbs. ***A***, Ultrathin sections from LR White-embedded mouse neocortex immunolabeled with anti-PSD-95 mAb K28/43 (green) and a reference anti-synapsin Ab (Cell Signaling #5297, magenta, top), or a reference anti-PSD-95 mAb (Cell Signaling #3450, magenta, bottom). Nuclei are labeled with DAPI (blue). To the right, examples of individual synapses are shown, with four serial sections through each. Synapses 1–3 are immunolabeled with K28/43 (green) and anti-synapsin Ab (magenta), and synapses 4–6 with K28/43 (green) and the reference anti-PSD-95 mAb (magenta). Syn., synapse. ***B***, Immunolabeling of human neocortical samples from biopsy or autopsy with the same K28/43 mAb. While K28/43 performs well on human biopsy tissue (left), it shows very sparse labeling on autopsy tissue (middle). However, a different mAb from the same project, K28/77, gives a specific and robust signal on human autopsy tissue (right). Autofluorescent lipofuscin granules, which are much more abundant in the human tissue are seen in the green channel, within the neuronal cytoplasm surrounding the nuclei. ***C***, Top, Mouse neocortex postfixed with osmium tetroxide and immunolabeled with an anti-PSD-95 mAb (green) and a reference anti-synapsin Ab (Cell Signaling #5297, magenta). K28/43 gives dense nonspecific label, but mAb K28/86 from the same project performs well in this preparation. ***C***, Bottom, Immunogold electron microscopy of mouse neocortex with K28/91, two serial sections are shown. Excitatory synapses, recognized by their asymmetric synaptic junction (magenta asterisk), have associated immunogold particles, whereas inhibitory synapses (cyan asterisk, symmetric synaptic junction) do not.

10.1523/ENEURO.0290-23.2023.f1-2 Extended Data Figure 1-2 Commonly used antibodies for AT, grouped by target. These antibodies have been validated in both mouse and human biopsy brain tissue, except where indicated (**). The great majority of antibodies perform well with both formaldehyde fixation and with a combination of formaldehyde and glutaraldehyde, except several that require glutaraldehyde in the fixative (*). RRID, Research Resource Identifier. Download Figure 1-2, DOCX file.

### Finding application-specific anti-PSD-95 mAbs via retrospective screen of a prior monoclonal project

We previously performed a project to develop mAbs recognizing the mammalian synaptic marker PSD-95 employing a region of human PSD-95 (amino acids 77–299 of Uniprot accession number P78352-2) as the immunogen. This resulted in a set of 96 independent samples that displayed immunoreactivity against a recombinant PSD-95 protein fragment by ELISA, and to varying degrees on immunoblots and by immunohistochemistry against brain samples (Tiffany et al., 2000; Rasband et al., 2002). From these 96 samples we selected one mAb, K28/43, that exhibited efficacy and specificity for reliable labeling of mammalian PSD-95 in brain tissue sections and cultured neurons. However, all 96 ELISA-positive samples had been archived as frozen hybridomas for potential future use.

In a subsequent analysis of mAbs from this project aimed at identifying mAbs recognizing zebrafish PSD-95, we observed that binding to mammalian PSD-95 was not predictive of labeling the zebrafish ortholog (Meyer et al., 2005). Whereas clone K28/43 robustly recognized mammalian PSD-95 (Rasband et al., 2002), it did not recognize zebrafish PSD-95, although other mAbs from this same project did (Meyer et al., 2005). Human and zebrafish PSD-95 (Uniprot accession number A0A8M3ASX4) share 89% amino acid identity within the region used as the immunogen, with distinct regions of high and low sequence identity. This provides a likely basis for mAbs with distinct epitopes within the collection originally selected for their binding to human PSD-95 displaying differences in binding to zebrafish PSD-95. That we were successful in rescreening existing mAbs within this collection for a new purpose suggested that this would be a viable approach to identify mAbs for not only new targets, but also for new applications, without the need to launch new mAb projects from scratch.

Accordingly, we evaluated mAbs for labeling of PSD-95 in samples processed for AT (Fig. 2*A*). Following the strategy outlined in Figure 1, controls to assess specificity included co-labeling with different reference Abs against the same target (a rabbit monoclonal anti-PSD-95 Ab, Cell Signaling #3450) and Abs against the adjacent presynaptic compartment of excitatory synapses (a rabbit monoclonal anti-synapsin Ab, Cell Signaling #5297). Because postsynaptic densities of synapses usually span at least two adjacent ultrathin sections (70-nm thickness each), the consistency of labeling was further assessed by examining serial sections for the presence of immunolabel. Using these criteria, we observed robust and specific labeling with K28/43 of excitatory synapses in AT sections prepared from mouse cortex, and from freshly obtained resected human neocortex (Fig. 2*B*). However, no specific labeling with K28/43 could be detected in neocortical samples from human autopsy brain, although the tissue was fixed and embedded using the same protocol. Therefore, we expanded our search to include other mAbs from the K28 anti-PSD-95 project that like K28/43 had been identified as labeling brain tissue prepared using conventional immunohistochemistry protocols. We found that unlike K28/43, mAbs K28/37-labeled, K28/74-labeled, and K28/77-labeled synapses in both fresh and autopsy human brain samples, as well as in mouse neocortex samples.

Variations in the preparation of the AT samples also affected the performance of the mAbs. Thus, while K28/43 labeled conventional mouse neocortex AT sections, it did not label AT sections after tissue treatment with osmium (Fig. 2*C*), a preparation condition commonly used to preserve ultrastructure and provide contrast for EM. However, mAbs K28/38-labeled, K28/74-labeled, K28/86-labeled, and K28/91-labeled osmium-treated tissue in AT sections, and also subsequently yielded specific immunogold labeling (Fig. 2*C*). Overall, immunogold labeling on osmium-treated tissue was not very efficient and usually resulted in low labeling density, prompting us to use a different method for tissue preparation for EM purposes, as detailed below. Results from these *post facto* analyses of an existing collection of PSD-95 mAbs illustrated that application-specific re-evaluation of mAbs can identify those with strong and specific labeling that may not be identified in other assays. In addition, they highlighted the potential for retrospective analyses of other archived mAb projects to identify mAbs with characteristics suitable for use in AT.

### Application-specific generation and validation of mAbs for AT

**Figure 3.**
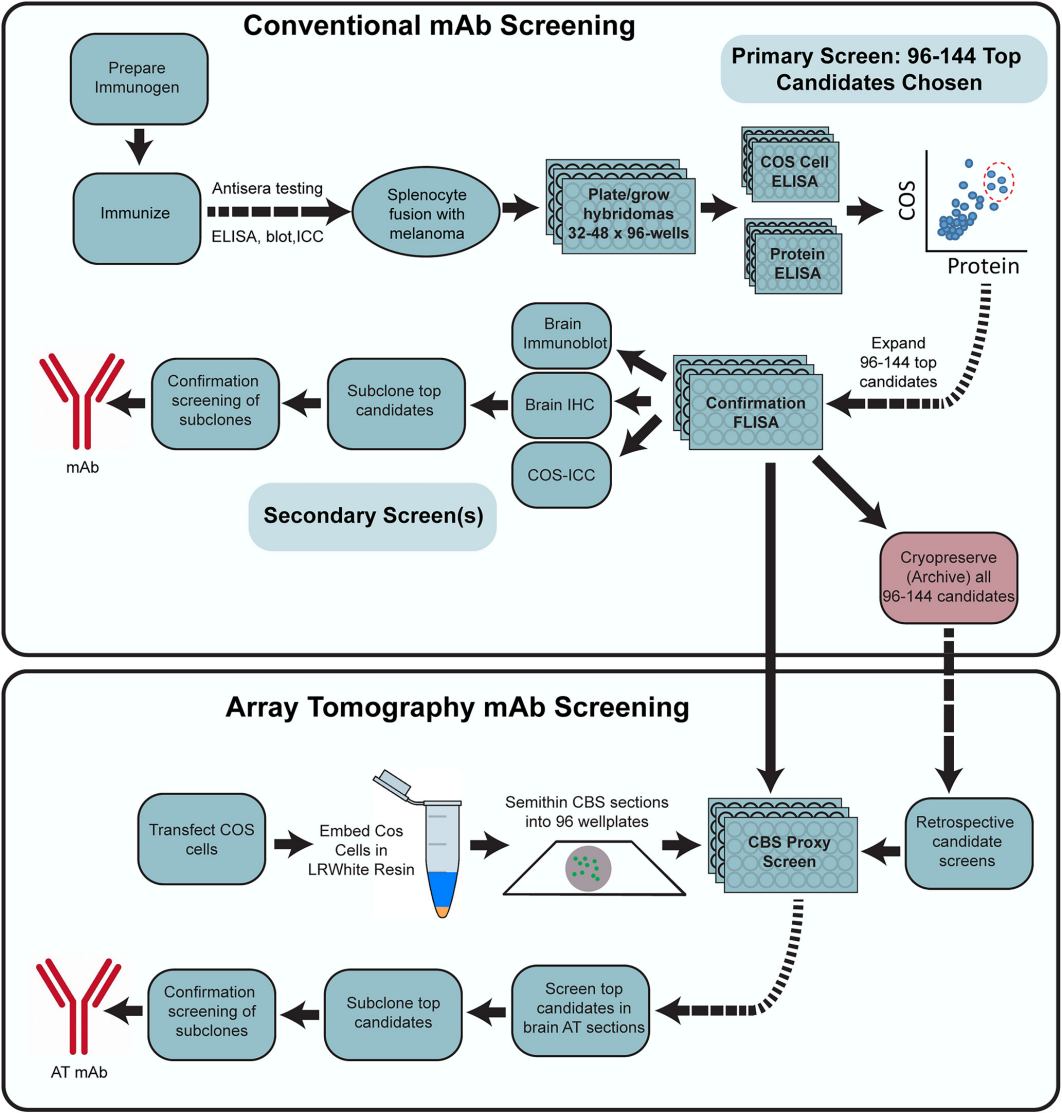
Flow diagram of the mAb screening workflow. Flow charts illustrating steps for conventional (top) and AT-inclusive (bottom) mAb screens.

Our generation of mAbs for neuroscience employs a stepwise screening workflow that incorporates the tissue culture aspects of classical hybridoma generation, expansion and archiving (immunization, hybridoma fusion, cell culture, cryopreservation) and parallel screening (ELISA, immunocytochemistry on transfected heterologous cells), while also including assays (immunoblots and immunohistochemistry) performed on mammalian brain samples (Trimmer et al., 1985; Bekele-Arcuri et al., 1996; Rhodes and Trimmer, 2006; Gong et al., 2016). The above experience in rescreening the PSD-95 mAb clones suggested that including samples prepared for AT would help identify mAbs useful for that application. To define AT-compatible mAbs, we first added an additional screen comprising immunolabeling and analysis of AT brain sections into our mAb pipeline (Fig. 3). However, we found that screening with AT on brain sections was too slow and labor intensive, given the large number of samples that needed to be prepared, immunolabeled, and evaluated. Moreover, developing an alternative cell-based proxy AT assay represented an opportunity to reduce the need for animal tissues. We therefore developed a rapid and straightforward cell-based proxy assay for mAbs able to recognize their target in samples prepared as for AT, employing transiently transfected cells as used in the immunocytochemistry screening step (Fig. 3, bottom).

### Homer1L mAb generation as an exemplar mAb project

**Figure 4.**
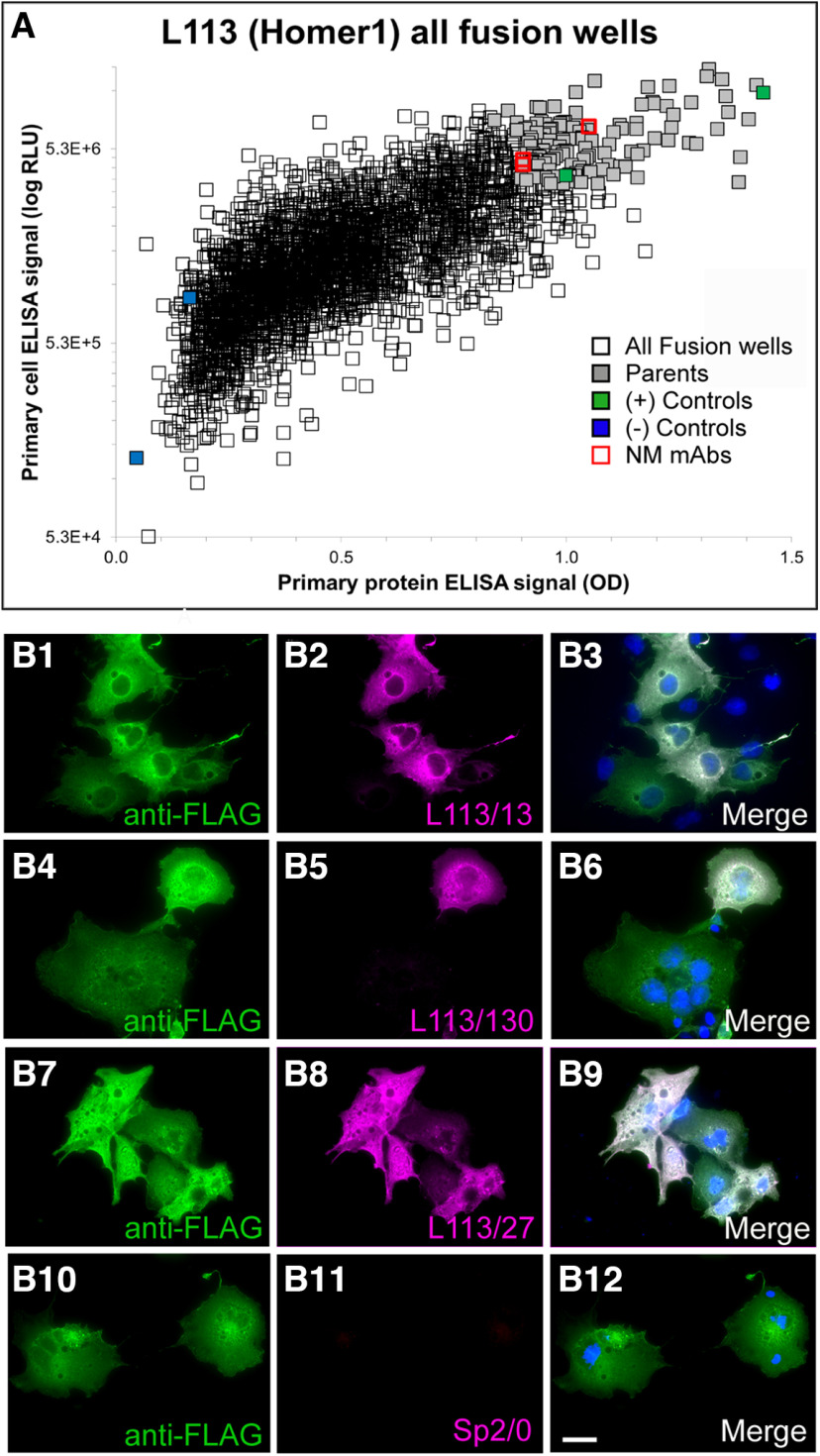
Conventional ELISA and COS-IF screening results for the L113 project targeting Homer1L. ***A***, ELISA primary screen data for the project, with protein and cell ELISA data plotted on the *x*- and *y*-axes, respectively. 2944 hybridoma samples were screened by two ELISA assays, which also included positive (green) and negative (blue) control wells. The 144 candidates selected for further screening are in gray, and the red squares denote the wells with candidates (L113/13, L133/27, L113/130) that were ultimately selected as NeuroMab mAbs. ***B***, Exemplar results of the secondary COS IF screen for the L113 project. Photomicrographs show fluorescent immunolabeling of COS-1 cells transiently transfected with Flag-tagged mouse Homer1L mammalian expression construct using a rabbit anti-Flag pAb (green; Sigma, catalog #F7425), candidate mouse mAbs (magenta) and Hoechst nuclear stain (blue). The first three rows show images from three positive candidates (L113/13, L113/27, L113/130 and) that were eventually selected as NeuroMabs, and the fourth row shows the negative control (Sp2/0 myeloma cell medium). Scale bar = 5 μm.

**Figure 5.**
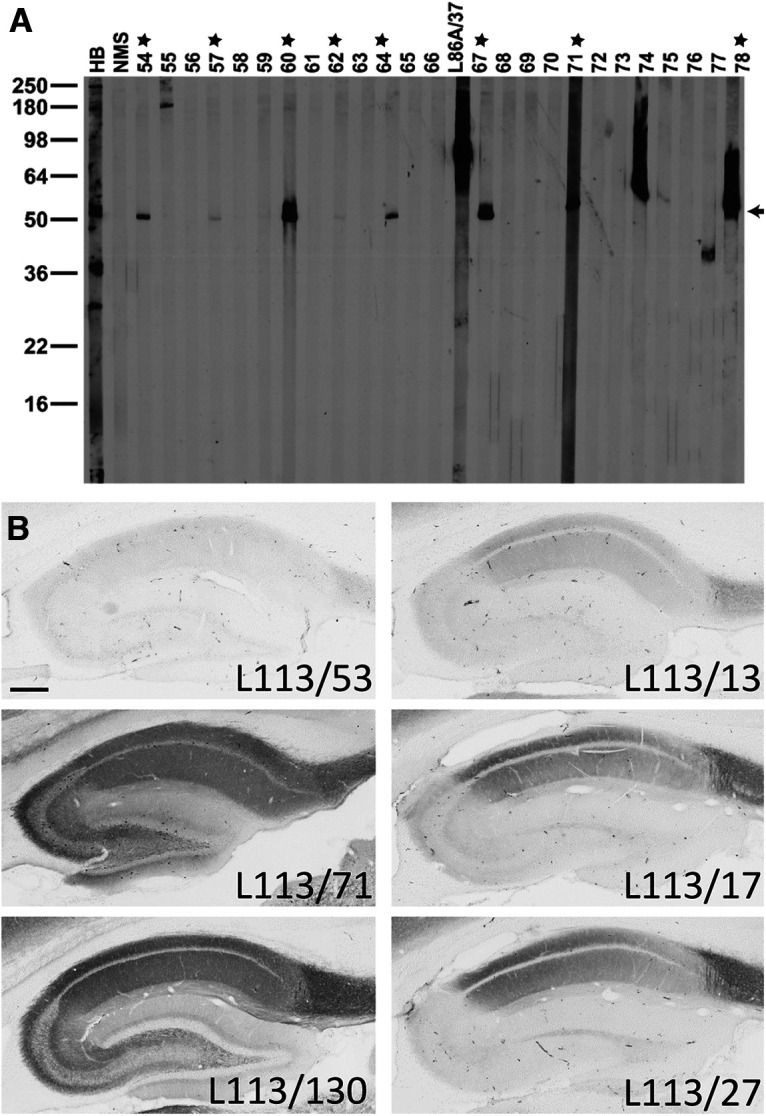
Conventional immunoblot and IHC screening results for Homer1. ***A***, Representative immunoblot strips from the L113 screen. Values on left show the mobility of molecular weight standards in kDa. Each lane represents a replicate strip containing a crude rat brain membrane fraction probed with a different candidate or control Ab. Other lanes include antiserum from one of the immunized mice (HB), a mouse serum negative control, and a positive control NeuroMab mAb against a different target (L86A/37, AMIGO-1). Strips for candidates L113/54 to L113/78 are shown. Stars = positive candidates on strip blot. Arrow = expected electrophoretic mobility of Homer1L. ***B***, Representative images from the L113 IHC screen. Photomicrographs show DAB/NAS immunolabeling of sagittal rat brain sections. Results from six candidate mAbs highlight a range of results from negative (L113/53) to partial (L113/17, L113/27, L113/13) to full (L113/71, L113/130) labeling, with the expected cellular and subcellular labeling pattern based on *in situ* hybridization and immunohistochemistry evidence gleaned from the literature and from publicly accessible *in situ* hybridization databases. Scale bar = 1 mm.

An exemplar mAb project (the L113 project) targeted Homer1L, an important component of the postsynaptic density of excitatory synapses (Xiao et al., 2000; Fagni et al., 2002; Brandstätter et al., 2004). We immunized a set of mice with a recombinant protein comprising the C-terminal two-thirds of the mouse Homer1L protein (amino acids 121–363 of accession number Q9Z2Y3-1), a primary sequence that is 97.8% identical to human Homer1L and 97.1% identical to rat Homer1L. This fragment contains an N-terminal region present in all Homer1 splice variants, and a C-terminal region unique to the longest splice variant Homer1L (Shiraishi-Yamaguchi and Furuichi, 2007). Next, we performed two sets of ELISAs on hybridoma conditioned culture medium (tissue culture supernatants; “TC supes”) harvested from individual wells of 32 × 96-well hybridoma culture plates. One set of ELISAs was against the purified fragment of Homer1L that was used to immunize the mice, and the other was against heterologous cells that had been transiently transfected to express the full-length mouse Homer1L protein and then fixed with 4% FA and permeabilized with 0.1% Triton X-100 (standard conditions for immunocytochemistry). We used the combined results from these two ELISAs to inform the selection of 144 hybridoma cultures for further screening, from the 2944 samples evaluated. A scatter plot comparing results from these two distinct Homer1L ELISAs is shown in Figure 4*A*; data points for the 144 candidates selected for expansion in tissue culture and further analysis in the screening workflow are shown in light purple. Samples of TC supes harvested from the expanded cultures of these 144 selected hybridoma samples were assayed in parallel for efficacy and specificity using fluorescent immunocytochemistry against transiently transfected heterologous cells expressing full-length Homer1L (Fig. 4*B*); immunoblots on brain samples (Fig. 5*A*); and DAB-HRP immunohistochemistry on fixed, free floating brain sections (Fig. 5*B*). The positive candidates from these assays represent distinct, partially overlapping subsets of the original 144 ELISA positive clones (Fig. 4), with different subsets of mAbs exhibiting efficacy in each assay. In parallel, we also subjected these 144 TC supes to a novel AT-specific cell-based proxy assay as described in the following section.

### Generating and validating mAbs for array tomography: AT-focused screening of an anti-Homer1L mAb project

Our standard mAb generation pipeline did not contain a single assay that reliably predicted which candidate mAbs would successfully label AT-prepared brain tissue (see above). Moreover, screening all ELISA-positive clones (typically 96 or 144) on conventional AT sections of plastic-embedded brain samples, which included manual scoring by trained observers, was excessively slow and labor-intensive. This was especially problematic because we strive to identify the best candidates for subcloning of the hybridoma cell cultures to monoclonality before their cryopreservation, which needs to occur within one week after the initial ELISA screen (Gong et al., 2016). Therefore, we developed a novel cell-based proxy screen with a high predictive value for mAbs that would ultimately prove to be effective on labeling brain tissue in plastic-embedded AT sections. We hypothesized that the major factor distinguishing antigenicity in AT brain sections from conventional IHC is the process (dehydration, resin infiltration, heat curing) involved in the embedding of AT samples in array plastic. Our standard mAb screening workflow employs immunofluorescence labeling of the target protein expressed in heterologous cells as an important screen (Figs. 3 and 4; Bekele-Arcuri et al., 1996; Gong et al., 2016). By employing transiently transfected cells, the samples assayed are a mosaic population of cells with high levels of target protein expression adjacent to nonexpressing cells. Since the identity of the transfected cell subpopulation is apparent by the use of an independent marker, it is easy to determine which candidates selectively label target-expressing cells. We predicted that plastic embedding of transiently transfected heterologous cells expressing target protein would provide a similarly quick, inexpensive, and effective screen for candidate TC supes that exhibit target protein labeling under AT conditions.

**Figure 6.**
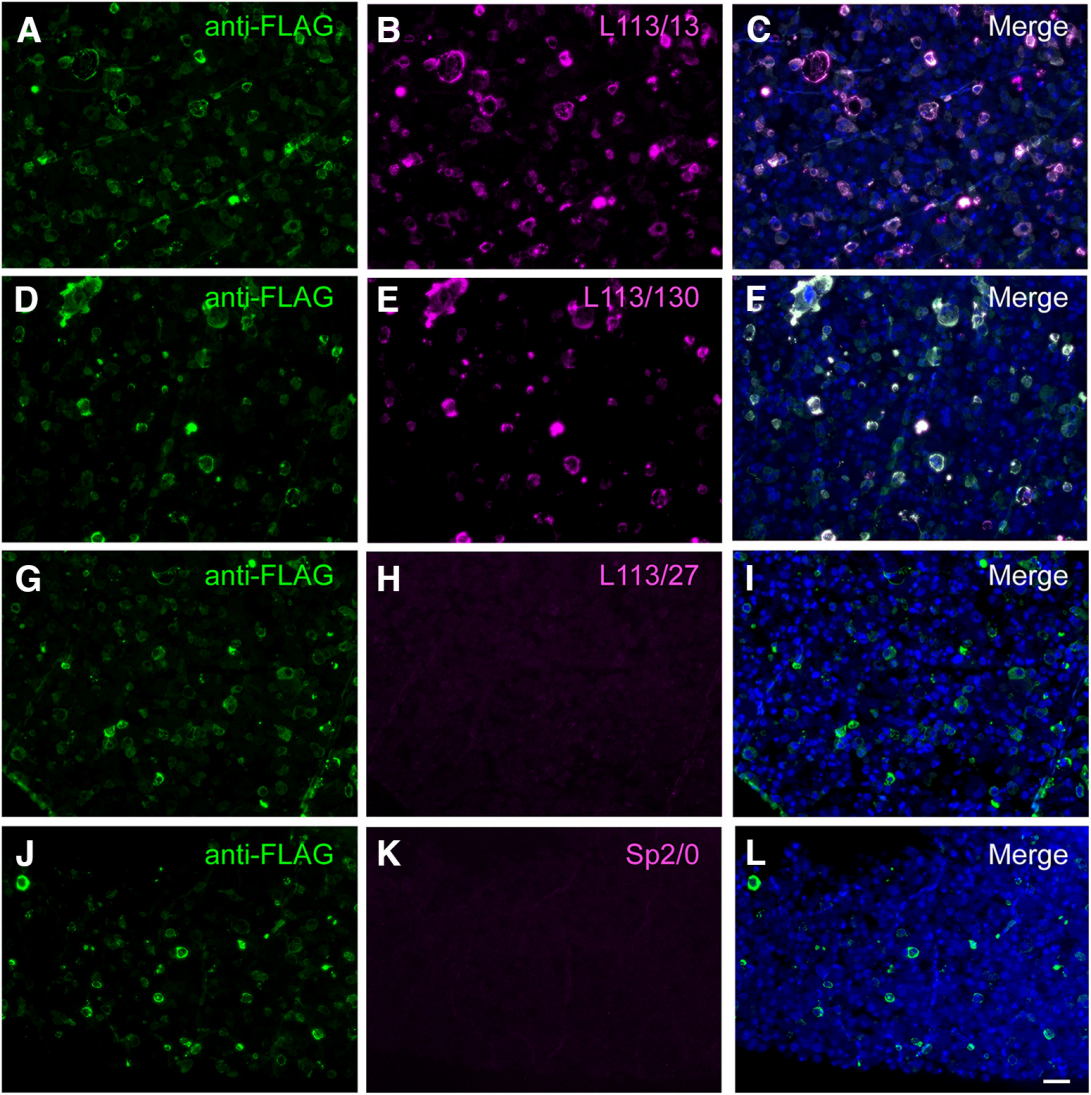
CBS assay identifies potential AT-compatible mAbs. Images of LR White embedded Homer1L-expressing transiently transfected COS-1 cells in semi-thin (400 nm) sections and labeled with candidate L113 mAbs. Only two of the three mAbs selected on the basis of their excellent performance in ELISA and conventional IHC screening were found to perform well on these AT proxy sections (L113/13; ***A–C*)** and L113/130 (***D–F***). mAb L113/27 does not selectively recognize the target expressing cells (***G–I***) and is similar in appearance to the negative control, conditioned medium from the Sp2/0 myeloma cell line (***J–L***). Scale bar = 50 μm.

For this cell-based screening assay, we transiently co-transfected heterologous COS-1 cells such that ≈50% of the cells were transfected to express both the target protein and an independent transfection “marker” to monitor transfection efficiency and to identify the transfected cells. Transfection markers were either encoded on separate plasmids (e.g., EGFP) co-transfected with target expression plasmids, or were encoded as tags fused to the target protein (e.g., an epitope tag or a fusion protein). For the CBS AT assay, transiently transfected cells were harvested 3 d after transfection, pelleted by centrifugation, fixed in suspension, re-pelleted and the fixed cell pellet embedded in LR White plastic. A portion of the same transfection cocktail was used to transfect cover slips containing cultured COS-1 cells that were subjected to conventional immunocytochemistry, to verify successful coexpression of both the marker and the target protein. Embedding of the cell pellet in AT plastic was performed using the same protocol as used for embedding brain tissue in plastic for AT (4% FA fixation, dehydration in an ethanol series, embedding in LR White resin and curing at 55°C for 24 h). Semithin (400 nm) sections that contained a mosaic of cells overexpressing the target protein and marker adjacent to cells devoid of target protein expression (Fig. 6) were cut and deposited into the wells of a collagen-coated clear bottom 96-well plate, which was used to screen up to 94 candidate TC supes (using the remaining two wells for positive and negative controls). The nucleus of each cell was labeled with Hoechst 33258, and bound primary Ab was detected using Alexa Fluor-594-conjugated anti-mouse IgG secondary Abs. Subsequent visual analysis was based on determining the presence of Alexa Fluor-594 signal and whether it was specific to the subset of cells with marker expression in green (Fig. 6).

### The AT CBS assay effectively predicts mAbs that label target proteins in AT brain sections

**Figure 7.**
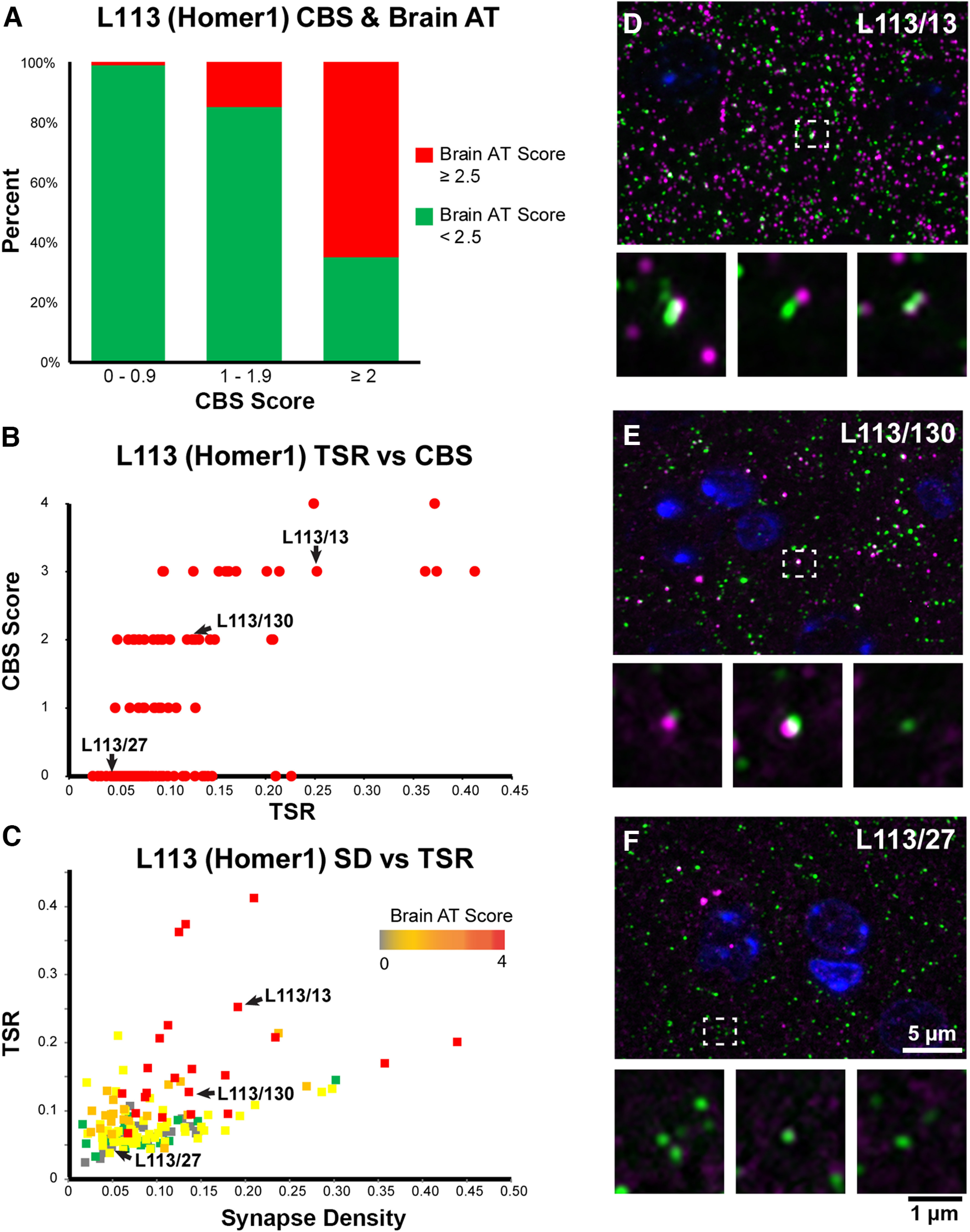
CBS positive mAbs screened on brain tissue embedded for AT. ***A***, Percent of candidate mAbs with high brain AT score among mAbs with different CBS scores. Candidate mAbs with low CBS scores (0–1 and 1–2) are very unlikely to have a high brain AT score, while the majority of candidate mAbs with high CBS scores also scored high on brain AT. ***B***, Correlation between TSR scores which measure Ab specificity in AT brain labeling, and CBS scores. ***C***, Target synapse density which measures the Ab sensitivity in AT brain labeling plotted against the TSR scores. ***D–F***, Images of ultrathin sections from LR White-embedded mouse neocortex immunolabeled with the Homer1L mAbs (magenta) L113/13 (***D***), L113/130 (***E***; both CBS positive), and the CBS negative L113/27 (***F***), double labeled with a PSD95 Ab (green). Nuclei are labeled with DAPI (blue). The bottom of each panel includes examples of individual synapses with three serial sections through each of the AT samples. Similar to their performance in the CBS assay (Fig. 5), mAbs L113/13 and L113/130 show specific labeling on AT brain sections, while L113/27 does not detect the target protein.

A proxy screen should be rapid, simple, and inexpensive, but most importantly it must be able to identify Abs that are effective when employed for their intended end use. We interrogated positive and negative samples from the AT CBS assay for labeling efficacy and specificity against brain samples in AT plastic. We found a high degree of concordance: positive samples from the AT CBS assay were much more likely to be scored as positives against AT brain sections than the population as a whole, and negatives from the CBS assays were rarely positive against AT brain sections (Fig. 7*A*). For example, in the exemplar Homer1L mAb screen, of the 144 candidate mAbs screened by CBS, 60% of the CBS-positive candidates (CBS score >2) gave good brain AT labeling (brain AT score >2.5), compared with only four out of 96 CBS-negative candidates (CBS score 0–1). To further assess the value of the AT CBS assay we used a previously described synaptic antibody characterization tool developed to quantitatively assess synapse Ab specificity in AT (Simhal et al., 2018). Using this tool we measured the target specificity ratio (TSR) which quantifies the ratio of target Ab label (e.g., Homer1L candidate mAbs) colocalizing with a reference synaptic marker, for example Abs against Synapsin-1 (Simhal et al., 2017, 2018). We observed a positive correlation between TSR scores for brain AT labeling and the respective CBS scores, supporting that the CBS assay was highly predictive in identifying brain AT compatible candidate mAbs (Fig. 7*B*). The target synapse density, i.e., the number of synapses detected with the antibody per unit volume reflects the sensitivity of the antibody, and was another metric used to characterize candidate antibodies. Antibodies with high brain AT score tend to have higher synapse density (Fig. 7*C*) and higher TSR as measured with the synaptic antibody characterization tool. Together these observations illustrate that the CBS assay can identify Ab samples with a high likelihood of successful brain AT labeling.

### Using the AT CBS assay to screen Abs for immunoelectron microscopy

During our initial screening of available polyclonal and monoclonal Abs against synaptic proteins for AT we had also observed that a high proportion of Abs effective for immunofluorescence AT on LR White-embedded sections also perform well on Lowicryl HM20-embedded sections (82%, 73 out of 89 Abs). Because Lowicryl embedding provides EM ultrastructure superior to that of LR White, we wondered whether the CBS AT assay could also screen for Abs effective for postembedding immunogold EM of brain samples in Lowicryl. We first tested whether the mAbs identified in the AT CBS screen performed on cells embedded in LR White plastic would also exhibit effective and specific immunofluorescence labeling of samples prepared using a protocol similar that to prepare samples for analysis by electron microscopy (brain samples fixed in FA and glutaraldehyde and embedded in Lowicryl plastic). A total of 17 positives from the CBS evaluation of samples prepared in LR White plastic were tested on AT sections of mouse neocortex fixed in FA and glutaraldehyde and embedded in Lowicryl plastic; of these, 12 (71%) were positive (Extended Data Fig. 1-1).

**Figure 8.**
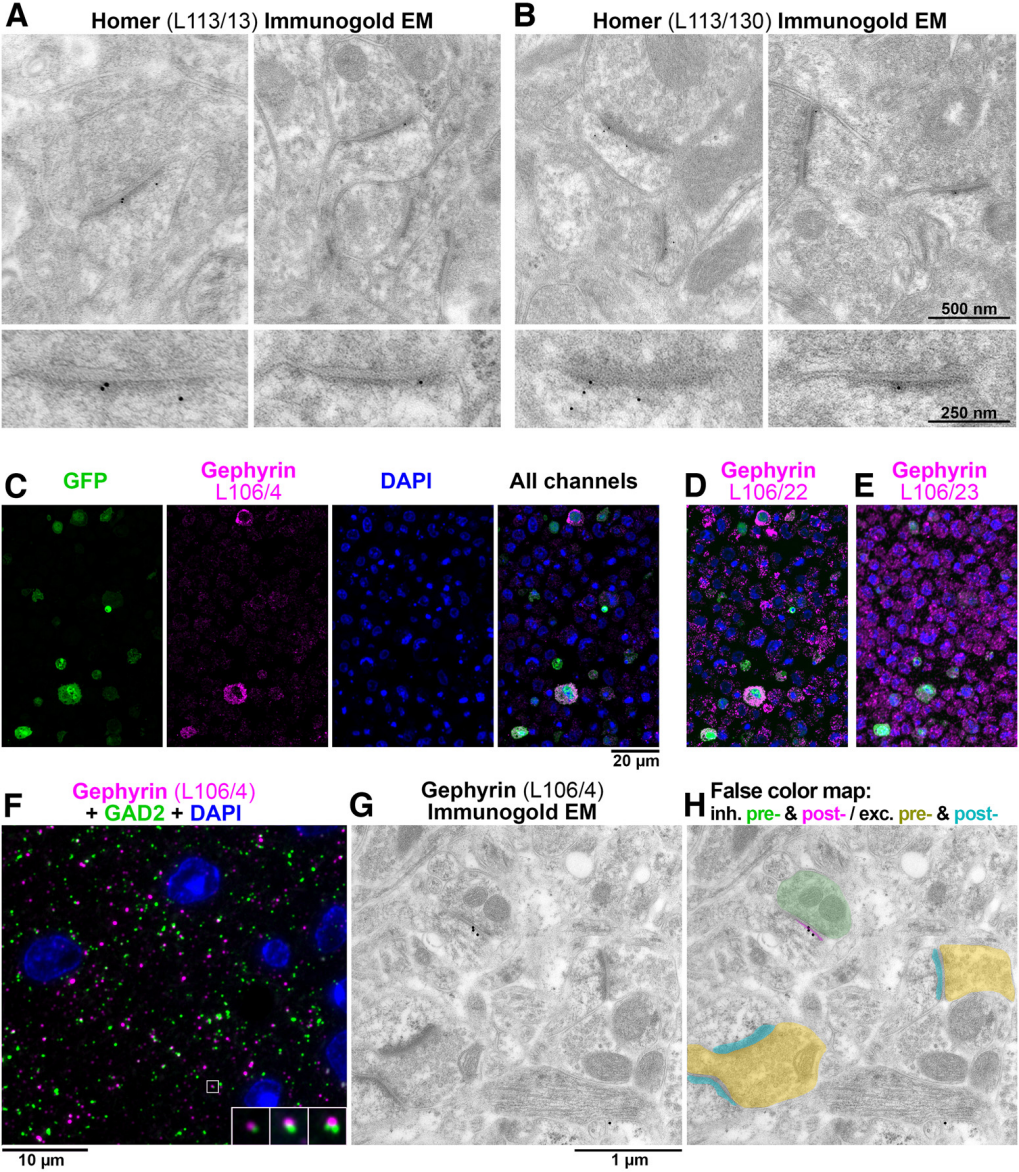
Ab screening for immunogold electron microscopy. ***A***, ***B***, Immunogold EM with L113/13 (***A***) and L113/30 (***B***) mAbs on Lowicryl HM20-embedded tissue from mouse cortex. The immunogold label localizes at asymmetric postsynaptic densities. The bottom panels show a magnified and rotated view of a postsynaptic density from the top panel. ***C***, AT CBS assay for gephyrin mAb L106/4 immunolabeling of a 400-nm section from COS-1 cells co-transfected with separate plasmids encoding gephyrin and EGFP and embedded in LR White. The gephyrin immunolabeling colocalizes at the cellular level with EGFP marker expression. Because in this case the COS-1 cells were co-transfected with two separate plasmids, the overlap is not complete and some GFP-positive cells do not label with L106/4. ***D***, An adjacent 400-nm section immunolabeled with mAb L106/22. While this mAb recognizes the transfected cells, there is also a high level of nonspecific labeling and therefore it was rejected. ***E***, mAb L106/23 does not label the transfected cells and was also rejected. ***F***, AT immunofluorescence of an LR White-embedded 70-nm section from adult mouse cortex with L106/4 mAb against gephyrin (magenta), rabbit mAb GAD2 (Cell Signaling #5843, green) and DAPI (blue). The insert shows three consecutive sections through the synapse that is marked with a white box. ***G***, Immunogold EM using the same L106/4 mAb on Lowicryl HM20-embedded tissue from mouse cortex. ***H***, False color map of the section in ***G***. The immunogold is associated with the postsynaptic side of the inhibitory synapse, but not excitatory synapses in the same field of view. See Extended Data Figures 8-1 and 8-2 for additional examples of Immunogold EM labeling.

Five of these mAbs was further evaluated at the EM level using immunogold labeling (Fig. 8). The performance of the antibodies was considered to be good if they labeled the expected target with a high ratio of signal-to-noise. Both of the Homer mAbs identified using the AT CBS assay (L113/13 and L113/130; Figs. 6 and 7) performed well when used for immunogold EM on Lowicryl HM20-embeded sections from mouse brain (Fig. 8*A*,*B*; Extended Data Figs. 8-1 and 8-2). Three different mAbs against gephyrin (a postsynaptic protein at inhibitory synapses) that were similarly identified using the AT CBS assay (Fig. 8*C–F*), were also confirmed as suitable for immunogold EM (Fig. 8*G*,*H*). Thus, the AT CBS assay of LR White embedded samples also has a high predictive value for identifying Abs effective for postembedding immunogold electron EM on brain tissue embedded in Lowicryl HM20.

10.1523/ENEURO.0290-23.2023.f8-1 Extended Data Figure 8-1 L113/13 immunogold EM on mouse tissue. Examples of L113/13 immunogold labeled synapses from mouse neocortex embedded in Lowicryl HM20. Download Figure 8-1, TIF file.

10.1523/ENEURO.0290-23.2023.f8-2 Extended Data Figure 8-2 L113/130 immunogold EM on mouse tissue. Examples of L113/130 immunogold labeled synapses from mouse neocortex embedded in Lowicryl HM20. Download Figure 8-2, TIF file.

### Application of the AT CBS assay to development of novel mAbs

**Figure 9.**
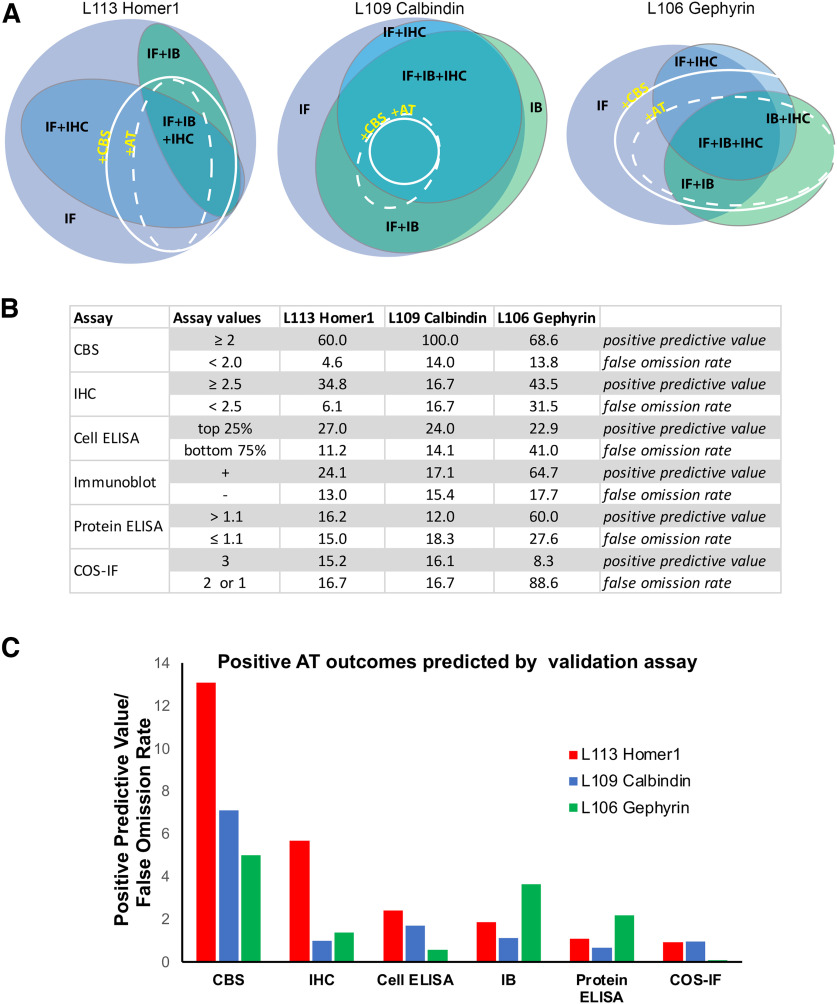
The CBS assay has high predictive value for Ab success in AT experiments. ***A***, Euler diagrams for projects L113 (Homer1), L109 (Calbindin), and L106 (Gephyrin). ***B***, Table listing the percent of ELISA positive candidates giving rise to brain section AT positive mAbs (score ≥ 2.5) broken down by their performance on each validation assay. ***C***, Ratio of the positive predictive value for AT suitable mAbs to the false omission rate shown for each validation assay. The CBS assay was most predictive for identifying brain AT positive mAbs. IF, immunofluorescence; IHC, immunohistochemistry; IB, immunoblot; AT, array tomography; CBS, cell-based proxy screen.

We have completed 15 separate mAb projects, each targeting a distinct protein in which we used the CBS proxy assay to identify candidate mAbs that are effective for AT-based imaging. More than 1900 samples were screened with the CBS assay, and 259 CBS positive parents (CBS score ≥2) were identified. Out of the CBS positive parents, 207 were subsequently tested for AT on brain sections, and 124 out of these (60%) were also positive for AT on brain sections. Compared with the other assays that we performed, the CBS assay had a higher predictive value for identifying candidate mAbs positive for AT on brain sections (Fig. 9). Thus, for the three projects (L113 Homer1, L109 Calbindin, and L106 Gephyrin) where all top ELISA positive candidates (Fig. 3) were selected for screening on every assay, the CBS screen had a higher positive predictive value for mAbs suitable for brain AT than any other assay, as well as a lower false omission rate. Positive predictive values were calculated as the number of candidates that were positive by both CBS assay and on brain AT (true positives), as a percent of all positive CBS candidates. False omission rate was calculated as the number of candidates negative on the CBS assay but positive for brain AT, as a percent of all the negative CBS candidates, and therefore reflects the likelihood of missing positive brain AT candidates (Fig. 9).

Overall, from the 15 projects, 12 yielded AT-validated mAbs (Extended Data Fig. 1-1). Two projects did not yield any positive candidates on the AT CBS screen, although conventional IHC positives were obtained from both projects. Another project (L125 targeting Synapsin-3) failed for reasons unrelated to AT, as all of the obtained candidate mAbs were found to exhibit cross-reactivity to Synapsin-1. To verify that we were not inadvertently excluding candidate mAbs with potential AT utility by applying the AT CBS as a filter, for the two projects that did not yield any AT CBS positive candidates we also tested a set of candidate mAbs that had high scores from the IHC screen, but none of these yielded labeling in the AT brain assay.

For many projects the CBS assay enabled us to develop more than one AT-validated mAb against the same target protein, either yielding mAbs of different mouse IgG subclasses (L106, L109, L122), or different target isoform specificity (L113, L127). These results support the reliability of the AT CBS assay for identifying a subpopulation of Abs that have a high likelihood of exhibiting labeling of their target in AT brain sections. The overall list of Abs tested and results can be found in Extended Data Figure 1-1.

## Discussion

The lack of highly validated Abs for research is a widely-recognized problem (Begley and Ellis, 2012; Baker, 2015a,b) that has forced laboratories to employ extensive in-house Ab testing before their use (Bordeaux et al., 2010; Micheva et al., 2010a). Here, we describe a systematic, rapid, and effective approach to validate Abs for brain AT, leading to a robust set of mAbs available to the research community (Extended Data Fig. 1-2). We introduce a simple and low-cost proxy assay with a high predictive value for Abs effective and specific for immunolabeling of AT brain sections. Unlike direct screening on AT brain samples, the cell-based proxy screen does not use samples from animals, reducing animal use. The visual analysis of the CBS assay is also more straightforward than AT screening. It utilizes a heterologous expression system, allowing specific candidate Ab labeling of transfected cells to be easily distinguished from nonspecific labeling of neighboring nontransfected cells. Visual comparison of transfected to nontransfected cells is much simpler than evaluation of AT brain samples, which must be performed at the level of individual synapses and may be confounded by synapse variability, low levels of expression or unknown distribution. Finally, because target proteins are overexpressed in transfected heterologous cells, the CBS assay is more sensitive at detecting Abs that may be of low concentration in hybridoma supernatants early in the development pipeline. This is particularly advantageous as the mAb candidate screening employs supernatants from hybridoma cultures, often with low levels of Ab, at a time in the development workflow when emphasis is on maintaining the health of the hybridoma cells before their cryopreservation, not on maximizing mAb accumulation in the medium. Moreover, at this stage hybridomas are not typically monoclonal; accordingly, these supernatants can contain multiple representations of target-specific mAb at very different concentrations, which may be lower than typically present in final mAb preparations after subcloning to monoclonality and growing under culture conditions designed to yield maximal mAb accumulation in the medium. Therefore, an elevated level of target protein expression facilitates successful labeling during early-stage screening.

In the experiments reported here, we included AT CBS screens in the workflow for 15 different projects, each aimed at developing mAbs against a distinct target. Each project employed screens aimed at developing mAbs for use in multiple downstream applications including transfected cell immunocytochemistry, brain immunoblots, immunohistochemistry on FA fixed conventional brain sections, and brain AT (Figs. 4 and 5). Comparing the outcomes with Euler diagrams (Micallef and Rodgers, 2014; https://www.eulerdiagrams.org/eulerAPE/), shows that performance in one of these applications does not predict success in other applications (Fig. 9), highlighting the need for application-specific screening. This confirmed our experience with many commercially available Abs, which were validated in applications other than AT, and often failed when used for AT.

Our results suggest that the false omission rate of the AT CBS assay relative to brain AT is quite low (≈5–15%). However, the AT CBS assay does yield “false positives” (candidate mAbs that work in the AT CBS assay but not for brain AT), likely because the AT CBS assays involve overexpression of the target protein in the transfected cells. Moreover, the use of heterologous cells means that the target protein may not undergo the same posttranslational modifications as it does in neurons, and proteins that interact with the target protein in neurons may not be expressed; accordingly, epitopes targeting posttranslational modifications or protein-protein interactions may be accessible in heterologous cells but not in brain samples. This is especially problematic for synapse-associated proteins, which are extensively regulated by posttranslational modifications and participate in complex and densely packed networks of interacting proteins (Grant, 2006, 2019; Zhu et al., 2018; Helm et al., 2021). This dense protein network can also result in more general Ab access problems, since access of the Ab to the synaptic compartment may be limited. Ineffective immunolabeling of synaptic targets because of these considerations led to the development of numerous protocols for enhancing Ab access in conventionally prepared brain sections (Watanabe et al., 1998; Fukaya and Watanabe, 2000). While ultrathin samples such as those prepared for AT are expected to have fewer issues with macro level Ab access to the synaptic compartment, there may still remain access problems at the molecular level, and these samples would presumably retain fixative-stabilized protein-protein interactions not present in heterologous cells, resulting in ineffective labeling of occluded epitopes at synapses but not in heterologous cells. Despite the occasional false-positives, the proxy CBS assay was effective at filtering out the numerous candidates that score as negatives in both cell-based and brain-based AT. In the exemplar Homer1 project there was a marked increase in the success rate in brain AT evaluation for those judged positive in the CBS assay (21/35 ≈ 60% of candidates with brain AT scores >2.5) compared with the success rate for the overall pool of 144 candidates (22/144 ≈ 15% of candidates with brain AT scores >2.5) that would have required evaluation had the CBS assay filter not been employed. Moreover, there was an extremely low false omission rate in the CBS assay (five candidates with a brain AT score >2.5 out of 109 candidates with CBS score ≤1.9). In contrast, other standard assays (conventional immunocytochemistry against transfected heterologous cells, immunoblots, conventional immunohistochemistry against brain sections) lacked predictive value for AT-effective Abs (Fig. 9).

Our results suggest that the same principles for Ab screening can be applied to other postembedding brain immunolabeling applications, including immunogold EM. In recent years, large-scale volume EM has provided important insights into the microscale organization of brain and principles of neuron connectivity (Zheng et al., 2018; Scheffer et al., 2020; Bae et al., 2021; Shapson-Coe et al., 2021). Complementing such expansive ultrastructural data with molecular information is rare (Anderson et al., 2011; Shahidi et al., 2015), due in large part to the lack of Abs suitable for postembedding immunoelectron microscopy. Our experiments suggest that the CBS proxy assay can also be used to identify Abs with high probability of success for immunogold EM, thus providing an efficient preliminary screen for suitable reagents for this highly demanding and resource-intensive application. Expanding the repertoire of synaptic Abs for electron microscopy applications will further increase the ability to collect correlated molecular and ultrastructural data in future connectomics studies. We suggest that this assay strategy could be employed whenever substantial collections of Abs against a given target need to be evaluated for AT or immunoelectron microscopy.

## Synthesis

Reviewing Editor: Matthew Grubb, King’s College London Decisions are customarily a result of the Reviewing Editor and the peer reviewers coming together and discussing their recommendations until a consensus is reached. When revisions are invited, a fact-based synthesis statement explaining their decision and outlining what is needed to prepare a revision will be listed below. The following reviewer(s) agreed to reveal their identity: Zoltan Nusser, Christopher Henstridge.

Reviewer 1:

The present MS describes a screening strategy to obtain antibodies that provide strong and specific labeling in resin embedded brain sections for array tomography (AT) and immunogold EM. This is clearly a critical aim if these techniques were to be more widely used methods in the fields of cellular/synaptic neuroscience.

There is a very important conclusion of the MS: different Abs are suitable for different sample preparations and therefore the concept that a single Ab is ‘good’ for every purpose is wrong.

One of the basic foundations of the MS is that the method, AT, has been optimized to an extent that no further improvement is (practically) possible. Given this as a starting point, indeed the authors are right that the only step in widening the use of AT is searching for and generating new Abs that work under this given condition. Theoretically, an alternative approach might be to develop the method further to make it appropriate for hundreds of already existing Abs. How do the authors know which approach would be more successful? One might argue that introducing e.g. a new retrieval step into the protocol could make 100 more Abs to provide strong, specific labelling. The reviewer feels that there is a general wisdom in the field that the method is limited by the number of available specific Abs. An alternative explanation is that the method needs to be optimized and once major improvements are made, many of the currently ‘bad’ Abs will turn to be brilliant.

There is a logical flaw in the method. The authors state that they cross-check the labeling of the tested Ab against the labeling of Abs that are known to provide specific and effective labeling in the tissue sample, such as PSD95 or synapsin1. This is very convincing in their mouse samples. However, already in the first test using human tissue, they claim that one of such ‘always working’ Ab (K28/43) does not provide a specific labeling in human autopsy sample. If this is possible then how do the authors know what is specific in human tissue and what not? Is the signal obtained with the ‘very good’ synapsin1 Ab specific? What is the evidence?

The authors argue that the CBS assay is ideal for screening a large number of Abs that will eventually be useful for AT. They argue that this method is faster than using plastic embedded brain tissue sections. However, it should be noted that separate CBS samples must be prepared for all target proteins, whereas a single brain section (one prepared block) contains all brain proteins. For screening, CBS is clearly faster, for sample preparation, it is not.

Page 28: ‘Both of the Homer mAbs identified using the AT CBS assay (L113/13 and L113/130, Figures 6 and 7) performed well when used for immunogold EM on Lowicryl HM20-embeded sections from mouse brain (Figure 8A, B).’ How was the quality of the immunogold reaction assessed? L113/13 Ab provided a single gold on one PSD and none on many other PSDs. Is that a good performance?

Minor points:

1) Page 7: ‘and embedded in LR White epoxy resin’. Epoxy?

2) It would be more elegant to show the same Ab for the AT and EM in figure 2C.

Reviewer 2:

This manuscript presents a useful pre-screening assay to increase the likely success of monoclonal antibodies for neuroscience, using the high-resolution imaging techniques array tomography and immunogold-EM. Furthermore, the authors provide a very useful extended table describing the antibodies they have screened and their success (or not) in AT staining of mammalian brain.

The manuscript is well written and contains clear descriptions of the methodology and results.

I only have a couple of minor points to highlight and a couple of questions, which are listed below in order of which they appear in the manuscript:

1 - Page 15 - It’s interesting to hear that K28/43 worked well on human biopsy material, but not autopsy material, despite the same tissue processing. However, other antibodies worked in both. Do the authors have any ideas from the different antibody epitopes for example, what differences may occur in the PSD-95 protein/structure/binding partners etc in the autopsy material to cause this difference?

2 - Page 16 Figure 2C - I couldn’t tell from the image or legend what the magenta label was, Synapsin or another PSD-95

3 - Page 23 - the authors state that the AT sections were scored manually, which is labour and time intensive. Would it not make sense to automate the screening for puncta overlap or direct opposition, potentially using the TSR mentioned later?

4 - Page 27 - Figure 7 - I don’t think Figure 7C is referred to in the text

5 - Page 29/30 - Figure 8 and legend - The Gephyrin L106/4 staining clearly doesn’t pick up all the GFP-positive cells and in the legend it is stated this is due to the transfection of 2 plasmids, so there wont necessarily be 100% overlap. One way round this for future screening, could be to use IRES-containing bicistronic vectors, containing GFP and target protein. That way both proteins are generated from the same plasmid in each cell.

6 - Discussion - Have the authors assessed other fixation methods, such as the glyoxal fix (https://www.science.org/doi/10.1126/sciadv.adf7084)? Could this help improve epitope access and increase AT success?

7 - General point - the vast majority of this work focuses on rodent brain analysis, but there is a growing number of researchers using AT for human analysis. It would be nice to mention how many antibodies have been tested on human tissue and with what success. I can see from the extended table there is a note if antibodies have been tested on mouse(M) and human(H), but the “worked on” column only states “yes” or “no”, with no indication if that means both or just mouse.

8 - Extended data table - This table will be a big draw for current AT users and i feel it could be tidied up a bit to be more user-friendly. For example, there is a blank “status” column, no indication what the “RRID” column is, “?” in some cells. To make things fool-proof, it might also be useful to colour code good ones, or highlight gold-standards for example.

Overall, this is a well written and nicely presented manuscript that will be of broad interest to the neuroscience field.

## Author Response

Response to reviewers

Please see our responses to each comment below. Reviewers’ text is in blue italic, and our responses in black. Thank you for helping us make this a better paper!

Reviewer 1:

“One of the basic foundations of the MS is that the method, AT, has been optimized to an extent that no further improvement is (practically) possible. Given this as a starting point, indeed the authors are right that the only step in widening the use of AT is searching for and generating new Abs that work under this given condition. Theoretically, an alternative approach might be to develop the method further to make it appropriate for hundreds of already existing Abs. How do the authors know which approach would be more successful? One might argue that introducing e.g. a new retrieval step into the protocol could make 100 more Abs to provide strong, specific labelling. The reviewer feels that there is a general wisdom in the field that the method is limited by the number of available specific Abs. An alternative explanation is that the method needs to be optimized and once major improvements are made, many of the currently ‘bad’ Abs will turn to be brilliant.”

The reviewer’s point is logically correct. Further refinement of existing protocols could enhance the labeling of other existing antibodies. This consideration motivated extensive work from our labs during the development of the AT technology, but we ultimately reached a “plateau,” leading us to conclude that substantial further improvement in the techniques was unlikely to yield much benefit.

The difficulty in further developing the method to make it more “antibody friendly” comes from two important considerations: First, because multiplexing is a major advantage of the method, one needs to find conditions that will be beneficial for all antibodies. Unfortunately, changing one parameter (e.g., gentler fixation) may improve the performance of an antibody, while decreasing the performance of other antibodies, or resulting in loss of smaller cytosolic antigens and thus hinder their detection.

Second, the ability to preserve ultrastructure and use both immunofluorescence and electron microscopy is a key feature of AT. Antigenicity can be improved (for example) by resin removal, but this can damage the ultrastructure, making it difficult to examine the tissue under the electron microscope. Reversing the order of imaging, using first EM and then immunofluorescence does not work either, because tissue antigenicity is lost after exposure to the electron beam. Previously we tried several published antigen retrieval protocols (e.g. protease treatment, citrate buffer) which in our hands did not lead to any noticeable improvement in antigenicity.

This doesn’t mean that optimization is impossible, but at this point we feel that finding approaches to develop more suitable antibodies may be more productive.

We have now added a clarification of this point in the Introduction, 2nd paragraph.

“There is a logical flaw in the method. The authors state that they cross-check the labeling of the tested Ab against the labeling of Abs that are known to provide specific and effective labeling in the tissue sample, such as PSD95 or synapsin1. This is very convincing in their mouse samples. However, already in the first test using human tissue, they claim that one of such ‘always working’ Ab (K28/43) does not provide a specific labeling in human autopsy sample. If this is possible then how do the authors know what is specific in human tissue and what not? Is the signal obtained with the ‘very good’ synapsin1 Ab specific? What is the evidence?”

We failed to make this clear in our original manuscript. We now explain that the PSD-95 reference antibody for both mouse and human (biopsy and autopsy) tissue is the Cell Signaling rabbit monoclonal PSD-95 (#3450). We have added this information to the Figure 2 legend where the human tissue labeling is shown, and a list of all reference antibodies is now included (Table 1 in the Methods section). All of the reference antibodies have been characterized extensively and used in several previously-published studies. All except gephyrin were evaluated using conjugate IF-SEM in mouse tissue (Collman et al. 2015); gephyrin was evaluated in the current manuscript. They were also validated using IF and the automated synaptic antibody characterization tool (Simhal et al. 2018), and by comparing synapse counts obtained with these antibodies vs. published EM studies (Simhal et al., 2019). In human biopsy tissue these antibodies give the same labeling pattern as in mouse, except that the synapse density in human cortex is much lower compared to mouse cortex and the synapses are larger. In human autopsy tissue the labeling is similar, but more variable, likely reflecting differences in patient health and in the tissue preservation. A study of synapses from human cortex is now in preparation.

“The authors argue that the CBS assay is ideal for screening a large number of Abs that will eventually be useful for AT. They argue that this method is faster than using plastic embedded brain tissue sections. However, it should be noted that separate CBS samples must be prepared for all target proteins, whereas a single brain section (one prepared block) contains all brain proteins. For screening, CBS is clearly faster, for sample preparation, it is not.”

The reviewer correctly notes that the CBS assay introduces an extra step into the screening process. However, in most cases this nevertheless proves advantageous for our task. The assay is ideal for screening a large number of Abs, where correct target labeling could be confounded by the complexity of brain proteome, and in cases where the distribution of the target protein is unknown or not well-characterized. Having a known single-protein target, as provided by the CBS assay, is a great advantage in such situations. This is now further clarified in the Discussion, 1st paragraph.

”Page 28: ‘Both of the Homer mAbs identified using the AT CBS assay (L113/13 and L113/130, Figures 6 and 7) performed well when used for immunogold EM on Lowicryl HM20-embeded sections from mouse brain (Figure 8A, B).’ How was the quality of the immunogold reaction assessed? L113/13 Ab provided a single gold on one PSD and none on many other PSDs. Is that a good performance?”

The total amount of label is highly sensitive to concentration of primary antibody. “Good performance” is perhaps best reflected by the ratio of signal to noise. We have now added two Supplemental figures with more images of Homer immunogold labeling (Figure 8-1: L113/13, and Figure 8-2: L113/130) to further illustrate this point. Immunonegative synapses are hard to interpret, since a) it is unclear what fraction of synapses in fact contain Homer 1L, b) postembedding immunoreaction detects only antigen at the surface of the plastic, and cannot “see” antigen lying deep within the section, and c) absence of label on one section does not exclude its presence on another section through the same synapse. Label efficiency is also inversely related the physical dimensions of the tag. We routinely use 10 nm gold because smaller particles are unduly difficult to visualize; note that standard fluorescent molecules are much smaller.

“Minor points:

1) Page 7: ‘and embedded in LR White epoxy resin’. Epoxy? “

Thank you for catching this mistake, now corrected.

“2) It would be more elegant to show the same Ab for the AT and EM in figure 2C. “

Agreed. Unfortunately, we don’t have publication quality pictures from the same antibody both for IF-AT and EM on tissue treated with osmium, as that screening was performed without a publication in mind.

Reviewer 2:

“1 - Page 15 - It’s interesting to hear that K28/43 worked well on human biopsy material, but not autopsy material, despite the same tissue processing. However, other antibodies worked in both. Do the authors have any ideas from the different antibody epitopes for example, what differences may occur in the PSD-95 protein/structure/binding partners etc in the autopsy material to cause this difference?”

The reviewer raises an interesting question. Unfortunately, we don’t understand the mechanistic basis underlying this antibody’s binding to its target to adequately address this.

“2 - Page 16 Figure 2C - I couldn’t tell from the image or legend what the magenta label was, Synapsin or another PSD-95”

Thank you for pointing this out. Synapsin is in magenta; this is now explained in the legend.

“3 - Page 23 - the authors state that the AT sections were scored manually, which is labour and time intensive. Would it not make sense to automate the screening for puncta overlap or direct opposition, potentially using the TSR mentioned later?”

We have used this approach in some of our antibody screens, however even the automated scoring of antibody performance on brain sections is much more labor-intensive than the scoring of the CBS. Calculating the TSR requires collection of images from at least 3 serial sections, which must be aligned and then analyzed using the computational tool.

“4 - Page 27 - Figure 7 - I don’t think Figure 7C is referred to in the text”

We now include a discussion of this figure panel in the text in Results, The AT CBS assay effectively predicts mAbs that label target proteins in AT brain sections.

“5 - Page 29/30 - Figure 8 and legend - The Gephyrin L106/4 staining clearly doesn’t pick up all the GFP-positive cells and in the legend it is stated this is due to the transfection of 2 plasmids, so there wont necessarily be 100% overlap. One way round this for future screening, could be to use IRES-containing bicistronic vectors, containing GFP and target protein. That way both proteins are generated from the same plasmid in each cell.”

The reviewer makes a good point. We will consider this approach in future screens.

“6 - Discussion - Have the authors assessed other fixation methods, such as the glyoxal fix (https://www.science.org/doi/10.1126/sciadv.adf7084)? Could this help improve epitope access and increase AT success?”

We have never tested glyoxal, but have in the past experimented with a variety of non-standard fixatives in an effort to enhance labeling. Most of the data from the cited reference, including the EM of Fig 3, rely on pre-embedding techniques, in which impaired antibody penetration into tissue is an important consideration. This is far less consequential for postembedding methods, as supported by the rather modest quantitative enhancement documented in Fig 5 of that publication. Long experience has persuaded us that weak fixation does little to improve postembedding immunohistochemistry. Though some epitopes are denatured with glutaraldehyde, some antigens (e.g. GABA) require the strong crosslinkage provided by glutaraldehyde to be detectable in tissue, and the ultrastructure is also markedly superior. We thus use two different fixatives, depending on the experimental requirements. Whenever the tissue will be examined in the electron microscope or preservation of GABA or other small molecules is required, we use a combination of formaldehyde and glutaraldehyde. For strictly light microscopic observations without the need for GABA preservation, we use formaldehyde only.

“7 - General point - the vast majority of this work focuses on rodent brain analysis, but there is a growing number of researchers using AT for human analysis. It would be nice to mention how many antibodies have been tested on human tissue and with what success. I can see from the extended table there is a note if antibodies have been tested on mouse(M) and human(H), but the “worked on” column only states “yes” or “no”, with no indication if that means both or just mouse.”

See response to point 8.

“8 - Extended data table - This table will be a big draw for current AT users and i feel it could be tidied up a bit to be more user-friendly. For example, there is a blank “status” column, no indication what the “RRID” column is, “?” in some cells. To make things fool-proof, it might also be useful to colour code good ones, or highlight gold-standards for example.”

These (7 and 8) are important points about making the antibody information more user-friendly. We now include an additional table with the more commonly used antibodies for AT of brain tissue (Figure 1-2), and we have also simplified the Extended data table with all the antibodies (Figure 1-1). We have clarified the abbreviations (RRID - Research Resource Identifier, now required by many journals to ensure transparency and reproducibility) and the “Tested In” column (the yes or no label applies to all species where the antibody was tested, unless explicitly stated).
