# Supplementary figures and images for: Developing a Toolbox of Antibodies Validated for Array Tomography-Based Imaging of Brain Synapses
Source: eNeuro. 2023 Dec 8;10(12):ENEURO.0290-23.2023. doi: 10.1523/ENEURO.0290-23.2023 (PMC10748464; doi:10.1523/ENEURO.0290-23.2023)

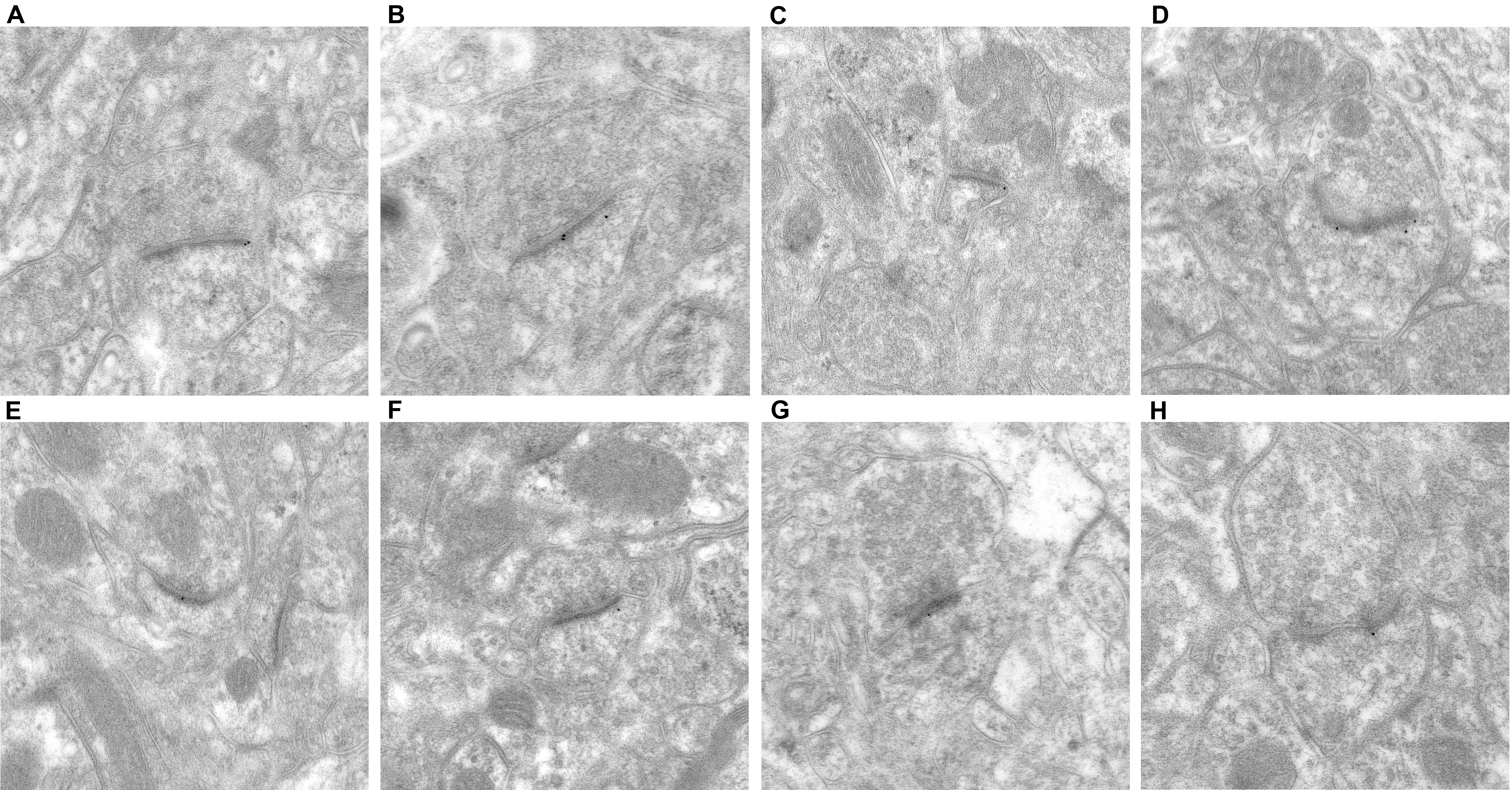

Supplement: Extended Data Figure 8-1 — L113/13 immunogold EM on mouse tissue. Examples of L113/13 immunogold labeled synapses from mouse neocortex embedded in Lowicryl HM20. Download Figure 8-1, TIF file. [file enu-eN-MNT-0290-23-s03.tif]

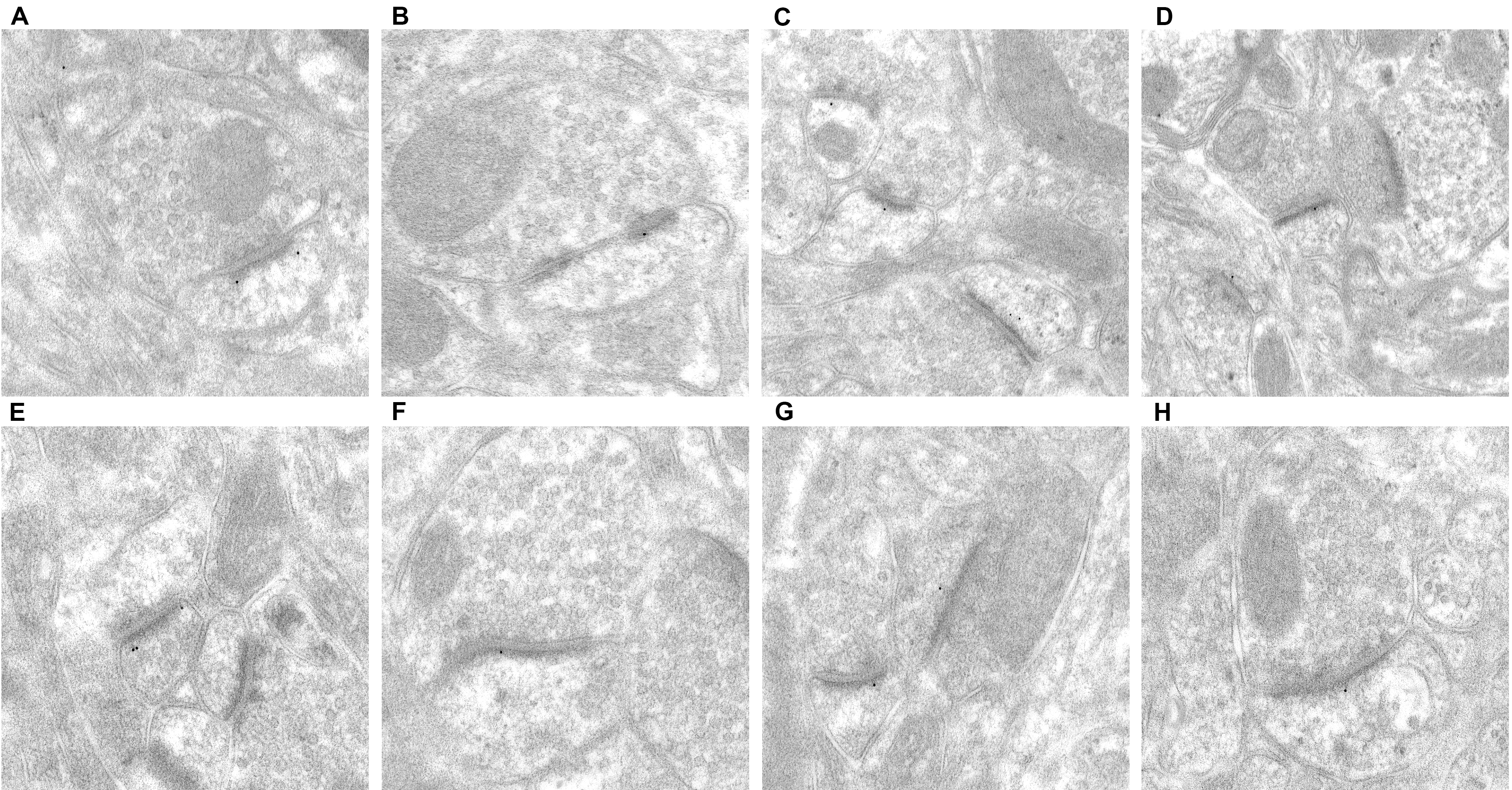

Supplement: Extended Data Figure 8-2 — L113/130 immunogold EM on mouse tissue. Examples of L113/130 immunogold labeled synapses from mouse neocortex embedded in Lowicryl HM20. Download Figure 8-2, TIF file. [file enu-eN-MNT-0290-23-s04.tif]
